# Isolation and Characterization of Distinct Rotavirus A in Bat and Rodent Hosts

**DOI:** 10.1128/jvi.01455-22

**Published:** 2023-01-12

**Authors:** Mai Kishimoto, Masahiro Kajihara, Koshiro Tabata, Yukari Itakura, Shinsuke Toba, Seiya Ozono, Yuko Sato, Tadaki Suzuki, Naoto Ito, Katendi Changula, Yongjin Qiu, Akina Mori-Kajihara, Yoshiki Eto, Hayato Harima, Daniel Mwizabi, Bernard M. Hang’ombe, William W. Hall, Ayato Takada, Yasuko Orba, Hirofumi Sawa, Michihito Sasaki

**Affiliations:** a Division of Molecular Pathobiology, International Institute for Zoonosis Control, Hokkaido University, Sapporo, Japan; b Division of Global Epidemiology, International Institute for Zoonosis Control, Hokkaido University, Sapporo, Japan; c Drug Discovery and Disease Research Laboratory, Shionogi & Co., Ltd., Osaka, Japan; d Department of Pathology, National Institute of Infectious Diseases, Tokyo, Japan; e Joint Graduate School of Veterinary Sciences, Gifu University, Gifu, Japan; f Department of Para-clinical Studies, School of Veterinary and Medicine, University of Zambia, Lusaka, Zambia; g Division of International Research Promotion, International Institute for Zoonosis Control, Hokkaido University, Sapporo, Japan; h Laboratory of Veterinary Public Health, Faculty of Agriculture, Tokyo University of Agriculture and Technology, Tokyo, Japan; i Department of National Parks and Wildlife, the Ministry of Tourism and Arts, Lusaka, Zambia; j Africa Center of Excellence for Infectious Diseases of Humans and Animals, University of Zambia, Lusaka, Zambia; k National Virus Reference Laboratory, School of Medicine, University College Dublin, Dublin, Ireland; l International Collaboration Unit, International Institute for Zoonosis Control, Hokkaido University, Sapporo, Japan; m Global Virus Network, Baltimore, Maryland, USA; n Department of Disease Control, School of Veterinary Medicine, the University of Zambia, Lusaka, Zambia; o One Health Research Center, Hokkaido University, Sapporo, Japan; p Institute for Vaccine Research and Development (HU-IVReD), Hokkaido University, Sapporo, Japan; Cornell University

**Keywords:** Rotavirus A, bats, rodents, genetic diversity, glycan specificity, human intestinal epithelium model

## Abstract

Rotavirus A (RVA) causes diarrheal disease in humans and various animals. Recent studies have identified bat and rodent RVAs with evidence of zoonotic transmission and genome reassortment. However, the virological properties of bat and rodent RVAs with currently identified genotypes still need to be better clarified. Here, we performed virus isolation-based screening for RVA in animal specimens and isolated RVAs (representative strains: 16-06 and MpR12) from Egyptian fruit bat and Natal multimammate mouse collected in Zambia. Whole-genome sequencing and phylogenetic analysis revealed that the genotypes of bat RVA 16-06 were identical to that of RVA BATp39 strain from the Kenyan fruit bat, which has not yet been characterized. Moreover, all segments of rodent RVA MpR12 were highly divergent and assigned to novel genotypes, but RVA MpR12 was phylogenetically closer to bat RVAs than to other rodent RVAs, indicating a unique evolutionary history. We further investigated the virological properties of the isolated RVAs. In brief, we found that 16-06 entered cells by binding to sialic acids on the cell surface, while MpR12 entered in a sialic acid-independent manner. Experimental inoculation of suckling mice with 16-06 and MpR12 revealed that these RVAs are causative agents of diarrhea. Moreover, 16-06 and MpR12 demonstrated an ability to infect and replicate in a 3D-reconstructed primary human intestinal epithelium with comparable efficiency to the human RVA. Taken together, our results detail the unique genetic and virological features of bat and rodent RVAs and demonstrate the need for further investigation of their zoonotic potential.

**IMPORTANCE** Recent advances in nucleotide sequence detection methods have enabled the detection of RVA genomes from various animals. These studies have discovered multiple divergent RVAs and have resulted in proposals for the genetic classification of novel genotypes. However, most of these RVAs have been identified via dsRNA viral genomes and not from infectious viruses, and their virological properties, such as cell/host tropisms, transmissibility, and pathogenicity, are unclear and remain to be clarified. Here, we successfully isolated RVAs with novel genome constellations from three bats and one rodent in Zambia. In addition to whole-genome sequencing, the isolated RVAs were characterized by glycan-binding affinity, pathogenicity in mice, and infectivity to the human gut using a 3D culture of primary intestinal epithelium. Our study reveals the first virological properties of bat and rodent RVAs with high genetic diversity and unique evolutional history and provides basic knowledge to begin estimating the potential of zoonotic transmission.

## INTRODUCTION

Rotavirus A (RVA) is the leading cause of diarrheal disease in young animals. In humans, RVA is responsible for >120,000 deaths/year in infants <5 years of age and children, mainly in developing countries (1). Although there is no specific treatment for RVA infection, two oral live attenuated vaccines (Rotarix and RotaTeq) have been recommended by the World Health Organization and are available in 114 countries (2). While these vaccines have reduced the number of hospitalizations and deaths due to RVA infection, atypical RVAs with high genetic diversity and genetic reassortment have been occasionally described and their origin should be monitored (3, 4).

RVA belongs to the family *Sedoreoviridae*, genus *Rotavirus*, which contains 9 species designated *Rotavirus A* to *Rotavirus J*. The RVA genome consists of 11 dsRNA segments, each encoding 6 structural viral proteins (VPs) and 5 or 6 nonstructural proteins (NSPs). RVA has a nonenveloped, triple-layered virion, with the outer capsid layer consisting of the spike protein VP4 and the glycoprotein VP7. VP4 and VP7 are traditionally used for genotype-based classification defining the P- and G-genotypes, respectively. Recently, a more comprehensive classification system based on the genotypes of all 11 segments has been proposed by the Rotavirus Classification Working Group (RCWG), defining the genotype constellation (GC) as follows: Gx-P[x]-Ix-Rx-Cx-Mx-Ax-Nx-Tx-Ex-Hx, representing each genotype of VP7-VP4-VP6-VP1-VP2-VP3-NSP1-NSP2-NSP3-NSP4-NSP5, respectively (5, 6). This classification and the accumulation of the whole genome sequence of RVA has facilitated our understanding of both the potential genetic diversity and genome reassortment events in RVA.

Although livestock such as pigs and cattle are reported to be a source of zoonotic transmission of RVA to humans, there have been limited reports involving wild animals (7–9). Rodents and bats are the largest and the second-largest orders of mammals, comprising about 40% and 20% of all classified mammal species in the world, respectively (10). In proportion to the number of species, they harbor a range of viruses, including zoonotic pathogens: coronavirus, henipavirus, lyssavirus, and filovirus in bats and hantavirus and arenavirus in rodents (11–17). As for bat RVAs, more than 30 strains have been identified worldwide in seven bat families (18–28). Even though some bat RVA genotypes are solely unique to bats, some genotypes are shared with human and other mammalian RVAs, indicating interspecies transmission and the zoonotic potential of bat-borne RVAs through genome reassortment events (18, 21–24, 26, 28). There has been considerably less research on rodent RVAs compared to bat RVAs. Some strains of mouse RVA were isolated before 1990 and have been used in experimental infections in laboratory mice (29). According to recent studies in Germany, China, and the United States, more than 15 RVA strains have been detected in wild rodents and shrews (30–33). Of note, Li et al. reported that rodent RVAs and human RVAs shared the same genotypes in some segments, indicative of local interspecies transmission (30). Most of these studies were based on genome detection and sequence analysis of RVA. For example, RVAs with bat-specific genotypes have not been isolated, despite the discovery of diverse bat RVAs. Further investigations which include virus isolation and investigation of the virological properties of bat and rodent RVAs, such as the cellular and host tropisms, transmissibility, and pathogenicity, are required to better characterize and confirm the zoonotic potential of animal RVAs.

Reverse transcription PCR (RT-PCR) with consensus primers has been widely used for RVA screening, but viral metagenomics have identified RVAs with diverse genomic sequences, which were not recognized by the screening primer sets (20). Recently, we reported that MA104-T2T11D cells exogenously transduced with human TMPRSS2 and TMPRSS11D, which belong to host type II transmembrane serine proteases, promote RVA infection in a trypsin-independent manner (34). The application of MA104-T2T11D cells for virus isolation offers high-throughput RVA screening in large numbers of field samples. In this study, we isolated RVAs with novel GCs from Zambian wild bats and rodents using MA104-T2T11D cells. Subsequently, we investigated the affinity of the identified RVAs for cell surface glycans, pathogenicity in suckling mice, and growth properties in an *ex vivo* model of human small intestinal epithelial cells.

## RESULTS

### Virus isolation-based RVA screening in wild animals.

**FIG 1:**
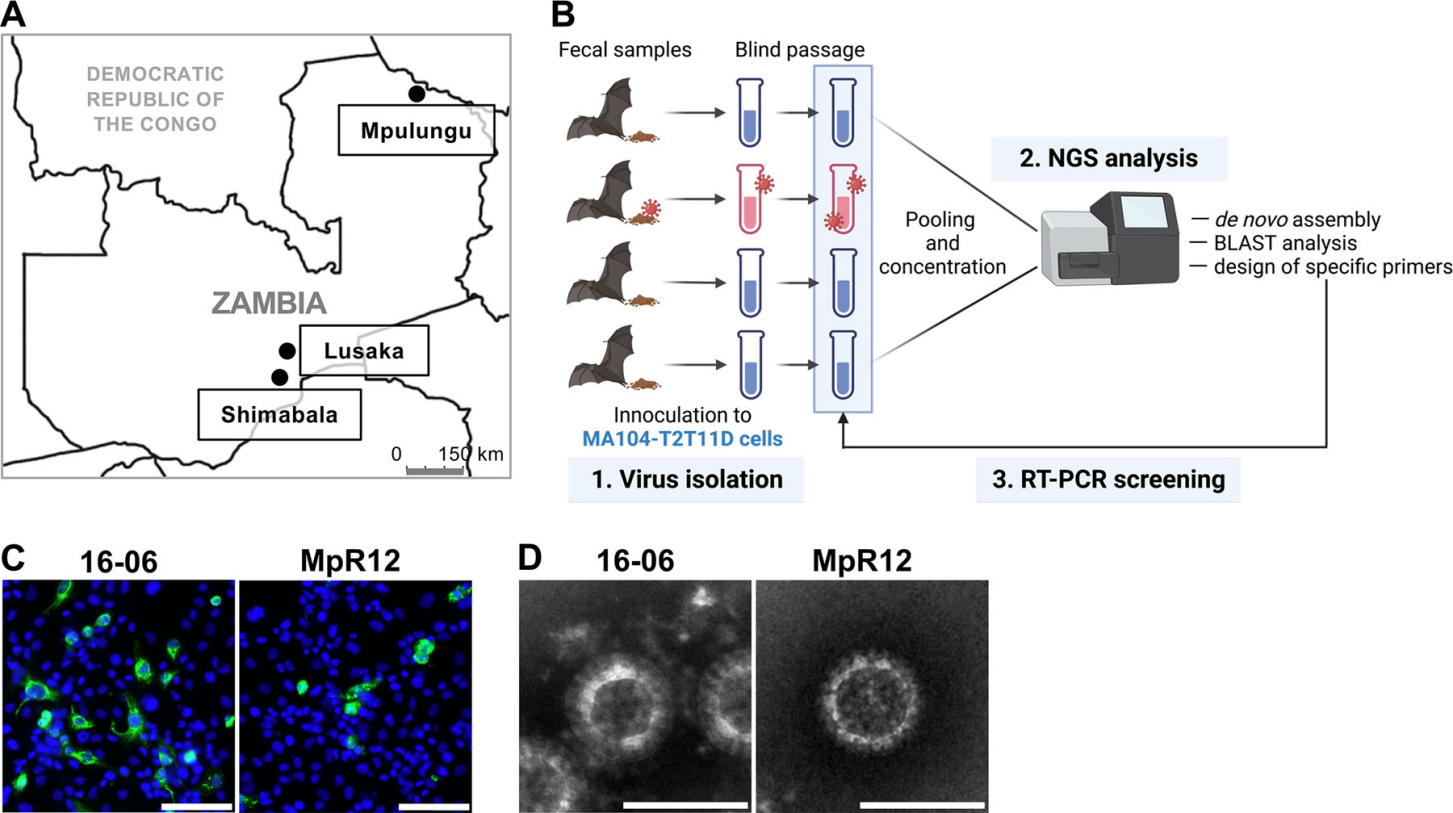
Isolation of rotavirus A (RVA) from wild animals in Zambia. (A) Map of sampling sites in Zambia. Egyptian fruit bats were captured in Lusaka and Shimabala, rodents and shrews were captured in Mpulungu. (B) Schematic workflow of virus isolation-based RVA screening. MA104-T2T11D cells were inoculated with fecal suspensions and cultured in roller tubes. After a single blind passage, the culture supernatants were pooled, concentrated, and analyzed by next-generation sequencing (NGS). If RVA genomes were detected, passaged culture supernatants were screened for RVA by reverse transcription PCR (RT-PCR) with specific primers for RVA sequences identified in the NGS analysis. Figure art was created with BioRender.com. (C) MA104-T2T11D cells infected with 16-06 and MpR12 were stained for RVA (green) and nuclei (blue). Scale bars = 50 μm. (D) Negative-stain electron micrographs of 16-06 and MpR12 virions. Scale bars = 100 nm.

To identify RVA from numerous samples, we initially employed virus isolation-based RVA screening. Feces or intestinal homogenates from 325 bats, 48 rodents, and 24 shrews in Zambia (Lusaka, Shimabala, and Mpulungu) were individually inoculated into MA104-T2T11D cells and cultured under rotary conditions (Fig. 1A and B). After a single blind-passage, the supernatants were pooled and examined for RVA using next-generation sequencing (NGS) (Fig. 1B). Based on the RVA sequences identified from the NGS data, we designed specific primers and screened each culture supernatant to identify the RVA. As a result, three strains of bat RVA, named 16-06, 16-27, and 18-12, were isolated from Egyptian fruit bats (Rousettus aegyptiacus) which were captured in Shimabala and Lusaka. Additionally, one rodent RVA strain, named MpR12, was isolated from a Natal multimammate mouse (Mastomys natalensis) which was captured in Mpulungu (Table 1 and Fig. 1A). After virus isolation, we investigated the prevalence of the isolated RVA strains by RT-PCR with general screening primers and specifically designed primers for the isolated strains (35). The RVA genome was detected exclusively from virus isolation-positive samples (Table 1).

**TABLE 1:** Sample information and the results of virus isolation

Species	Place	Yr	Virus isolation positive/total	RT-PCR screening positive/total	Isolated strain
Bat					
*Rousettus aegyptiacus*	Lusaka	2014	0/10	0/10	
		2015	0/178	0/178	
	Shimabala	2016	1/11 (9.1%)	1/11 (9.1%)	16-06
	Lusaka	2016	1/20 (5.0%)	1/20 (5.0%)	16-27
		2017	0/61	0/61	
		2018	1/45 (2.2%)	1/45 (2.2%)	18-12
Rodent					
*Mastomys natalensis*	Mpulungu	2012	1/28 (3.6%)	1/28 (3.6%)	MpR12
Other species^*a*^			0/20	0/20	
Shrew					
*Crocidura hirta*			0/23	0/23	
*Crocidura luna*			0/1	0/1	

a Other rodent species include *Aethomys chrysophilus*, *Rattus rattus*, *Cricetomys gambianus*, *Saccostomus* sp., *Steatomys* sp., *Grammomys* sp., *Gerbilliscus leucogaster*, and squirrels.

**FIG 2:**
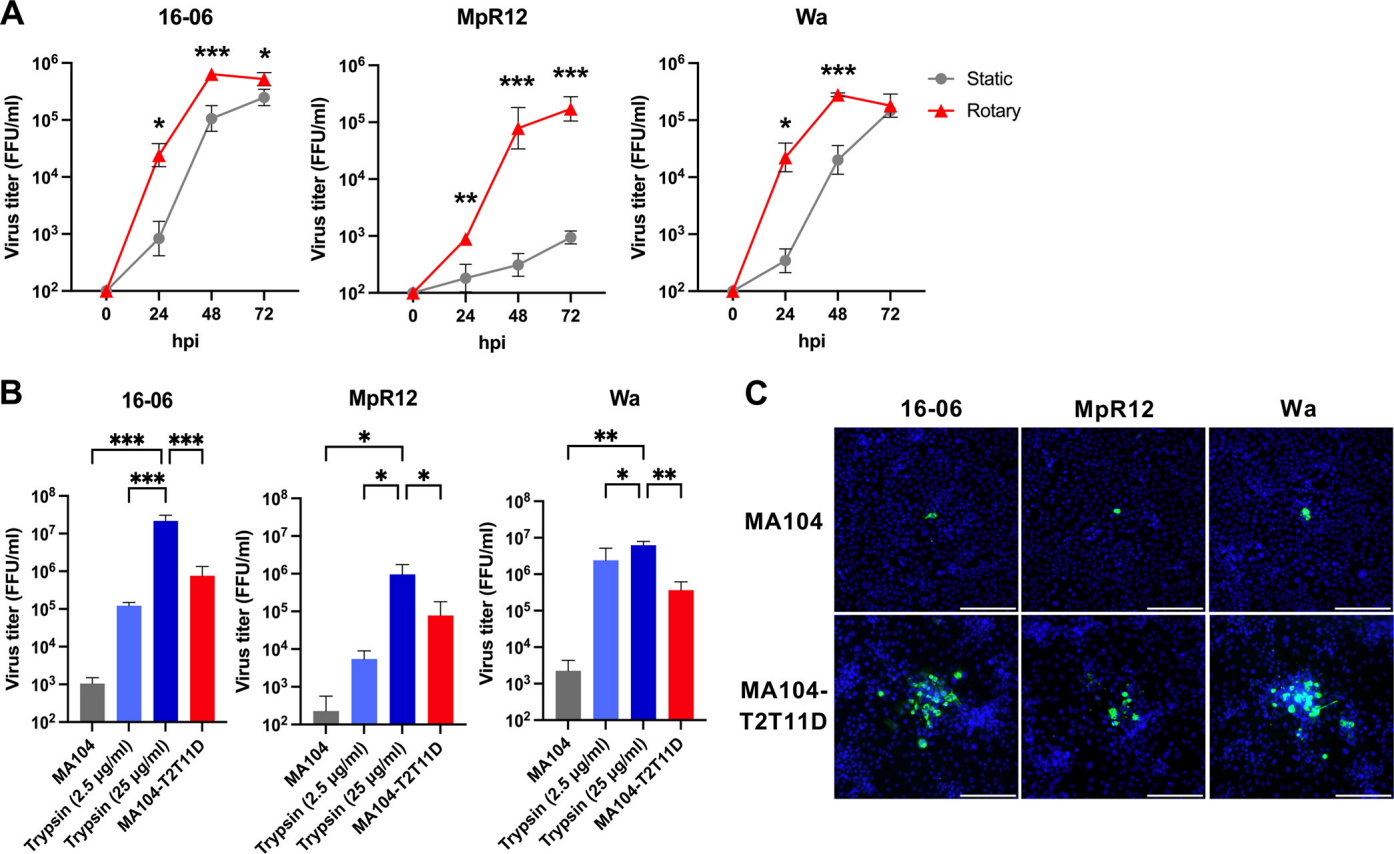
Growth kinetics of 16-06, MpR12, and Wa in different culture conditions. Monolayered cells were inoculated with 16-06 (MOI = 0.005), MpR12 (MOI = 0.1) and Wa (MOI = 0.1). Progeny virus in the supernatants was harvested at the indicated time points (hours postinfection [hpi]) and titrated by a focus assay. (A) Infected MA104-T2T11D cells were cultured in static and rotary culture conditions. (B) Viruses were infected and cultured in MA104, MA104 with trypsin (25 μg/mL or 2.5 μg/mL), or MA104-T2T11D cells. Virus titers at 48 hpi for each virus are given as means ± standard deviation (SD) of triplicate data from a representative experiment. Statistical analysis was performed using a Student’s *t* test (A) or one-way analysis of variance (ANOVA) with Tukey’s test (B). ***, *P *<* *0.001; **, *P *<* *0.01; *, *P *<* *0.05. (C) Representative focus induced by 16-06, MpR12 and Wa in MA104 cells and MA104-T2T11D cells. Infected cells were overlaid with 0.5% agar and cultured for 72 hpi. Fixed cells were stained for RVA (green) and nuclei (blue). Scale bars = 200 μm.

Because the three bat-derived RVA strains showed high nucleotide sequence identities (93.5% to 100% in each ORF; Table S1), 16-06 was chosen as a representative strain for subsequent analyses. Expression of antigens of 16-06 and MpR12 was validated by indirect immunofluorescence assays (IFAs) with an anti-RVA polyclonal antibody (Fig. 1C). Negative-stain electron microscopy identified 80- to 90-nm virus particles with wheel-like structures in the culture supernatants of the RVA-inoculated cells, which is the typical morphology of the RVA virion (Fig. 1D). To characterize the growth properties of the isolated strains, progeny virus titers in the supernatants were determined. Both 16-06 and MpR12 could be propagated in MA104-T2T11D cells, and their growth properties under rotary culture conditions were higher than those under static culture conditions (Fig. 2A). Notably, MpR12 showed limited growth in static culture, which is consistent with the growth characteristics of other RVA strains (36). To determine the trypsin dependency of the isolated strains, we examined virus titers at 48 h postinfection (hpi) in MA104 in the presence or absence of trypsin (Fig. 2B). Both 16-06 and MpR12 showed enhanced viral proliferation in a trypsin dose-dependent manner in MA104 cells (Fig. 2B). Virus titers of RVAs in MA104-T2T11D cells at 48 hpi were significantly higher than those in MA104 cells in the absence of trypsin (Fig. 2B). 16-06, MpR12, and Wa induced foci consisting of multiple cells in MA104-T2T11D cells, but not in MA104 cells, as described previously (Fig. 2C) (34). The foci formed by Wa and 16-06 were larger than those formed by MpR12 (Fig. 2C). These results indicate that the isolated 16-06 and MpR12 strains have different growth properties in MA104-T2T11D cells. In summary, infectious RVAs were successfully isolated from three Egyptian fruit bats and one Natal multimammate mouse from Zambia.

### Whole-genome sequencing and phylogenetic analysis of isolated RVAs.

**FIG 3:**
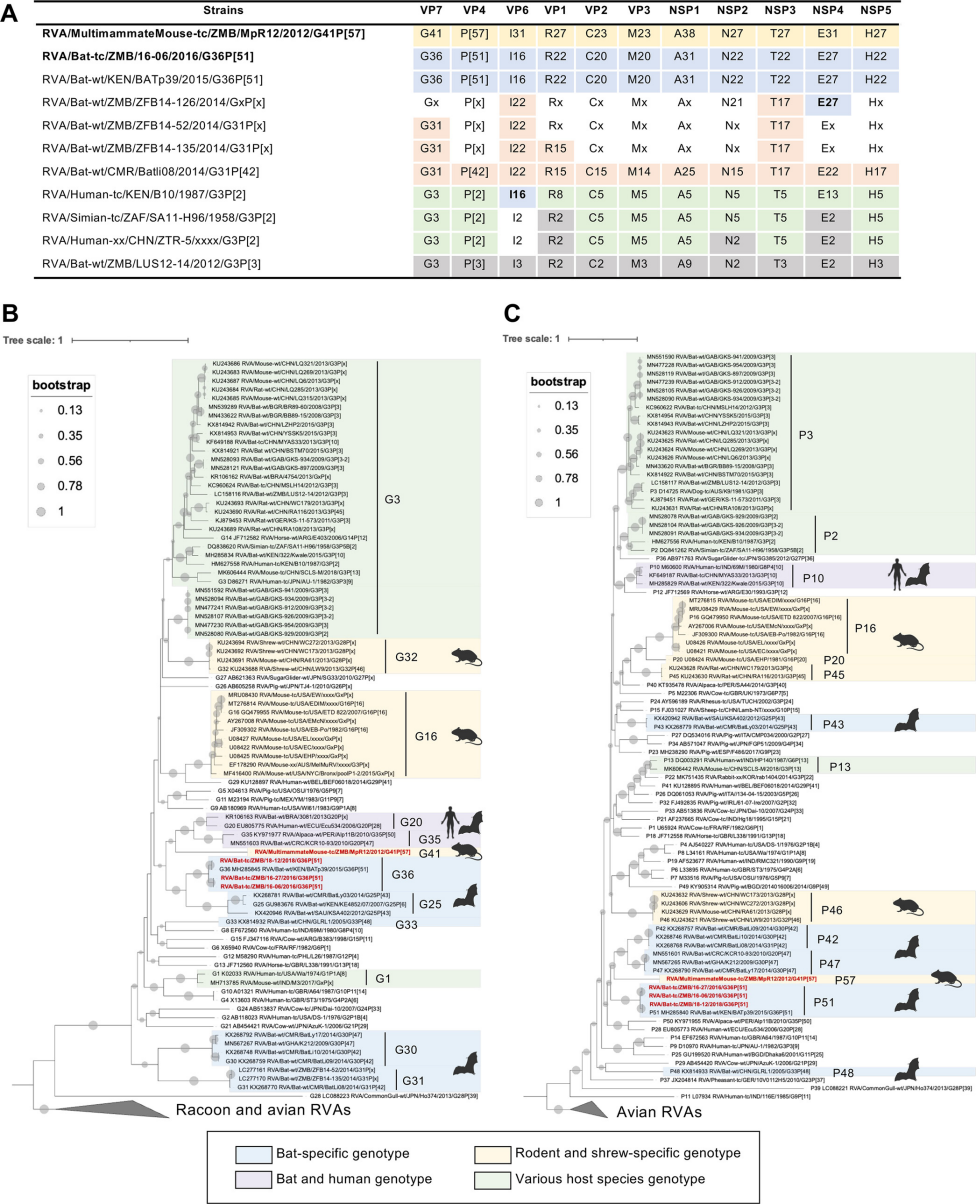
Whole-genome characterization of the isolated RVAs. (A) Comparison of genome constellations between the isolated RVAs and related RVA strains. Identical genotypes are shown in the same color, and genotypes of undetermined segments are indicated by “x.” (B and C) Maximum-likelihood tree of VP7 (B) and VP4 (C) genes based on the sequences of isolated RVAs, bat-derived RVAs, rodent-derived RVAs, and type strains of each genotype. Phylogenetic trees were constructed by the maximum-likelihood method using models of general time-reversible with gamma rate categories and invariant sites (GTR+G+I) with bootstrap values of 1,000 replicates. Avian and raccoon RVAs were regarded as the outer groups. The isolated RVAs are indicated in red. Bat-specific genotypes and rodent- and shrew-specific genotypes are highlighted in blue and yellow, respectively. Genotypes which include bat-derived and nontypical human RVAs are colored purple. Genotypes consisting of RVAs from multiple animal species are highlighted in green.

The complete open reading frame (ORF) sequences of all isolated RVAs (16-06, 16-27, 18-12, and MpR12) were obtained by *de novo* assembly of NGS sequence reads. In addition, the 5′ and 3′ untranslated regions (UTRs) of 16-06 and MpR12 were sequenced by rapid amplification of the cDNA end (RACE) method to determine the complete genome sequence. Both 16-06 and MpR12 had the typical genome size and structure of RVA, with the terminal sequences of the 5′ and 3′ UTRs broadly conserved. (Table S2) (37). Similar to other RVAs, 16-06 and MpR12 also encoded NSP5 and NSP6 in segment 11. The genome sequences of 16-06 and MpR12 were assessed using the Rotavirus A Genotype Determination tool to determine the GCs (Table 2) (38). The GC of 16-06 was G36-P[51]-I16-R22-C20-M20-A31-N22-T22-E27-H22, consisting of recently approved new genotypes and completely identical to that of RVA/Bat-wt/KEN/BATp39/2015/G36P[51] (BATp39), which is deposited as a bat RVA from *R. aegyptiacus* in GenBank but has not yet been published (Fig. 3A). In contrast, all segments of MpR12 showed high nucleotide sequence diversities with known RVAs, falling below the cutoff values for genotype assignment (5). Through consultation with RCWG, MpR12 was assigned to a new GC, G41-P[57]-I31-R27-C23-M23-A38-N27-T27-E31-H27, consisting of new genotypes (Fig. 3A). Finally, based on the nomenclature guideline of RCWG, the four isolated strains were formally named as RVA/Bat-tc/ZMB/16-06/2016/G36P[51] (GenBank accession no. LC704642 to LC704652), RVA/Bat-tc/ZMB/16-27/2016/G36P[51] (LC704653 to LC704663), RVA/Bat-tc/ZMB/18-12/2018/G36P[51] (LC704664 to LC704674), and RVA/MultimammateMouse-tc/ZMB/MpR12/2012/G41P[57] (LC638698 to LC638708) (6).

**TABLE 2:** Genotypes of all segments of isolated strain 16-06 and MpR12 with reference strains exhibiting the closest nucleotide identities

Strain	Gene	Genotype^*b*^	Strain exhibiting highest identity^*a*^			Cutoff (%)^*c*^
			Strain name	Accession no.	Nucleotide identity (%)	
RVA/bat-tc/ZMB/16-06/2016/G36P[51]	VP1	R22	RVA/bat-wt/KEN/BATp39/2015/G36P[51]_R22	MH285837	97.80	83
	VP2	C20	RVA/bat-wt/KEN/BATp39/2015/G36P[51]_C20	MH285838	93.77	84
	VP3	M20	RVA/bat-wt/KEN/BATp39/2015/G36P[51]_M20	MH285839	93.98	81
	VP4	P[51]	RVA/bat-wt/KEN/BATp39/2015/G36P[51]_P[51]	MH285840	94.89	80
	VP6	I16	RVA/bat-wt/KEN/BATp39/2015/G36P[51]	MH285841	97.24	85
	VP7	G36	RVA/bat-wt/KEN/BATp39/2015/G36P[51]_G36	MH285842	93.88	80
	NSP1	A31	RVA/bat-wt/KEN/BATp39/2015/G36P[51]_A31	MH285843	97.63	79
	NSP2	N22	RVA/bat-wt/KEN/BATp39/2015/G36P[51]_N22	MH285844	97.90	85
	NSP3	T22	RVA/bat-wt/KEN/BATp39/2015/G36P[51]_T22	MH285845	92.14	85
	NSP4	E27	RVA/bat-wt/KEN/BATp39/2015/G36P[51]	MH285846	97.92	85
	NSP5	H22	RVA/bat-wt/KEN/BATp39/2015/G36P[51]_H22	MH285847	98.16	91
RVA/MultimammateMouse-tc/ZMB/MpR12/2012/G41P[57]	VP1	**R27**	RVA/human-wt/RWA/669/2013/G1P[8]_R1	MN632939	72.73	83
	VP2	**C23**	RVA/dog-xx/USA/A79-10/1979/G3P[3]_C2	EU708935	76.49	84
	VP3	**M23**	RVA/human-wt/ZAF/2371WC/2008/G9P[8]_M1	JN013992	64.66	81
	VP4	**P[57]**	RVA/human-wt/UGA/MUL-12-117/2012/G3P[6]	KX655476	68.73	80
	VP6	**I31**	RVA/bat-wt/GHA/K212/2009/G30P[47]	MN567266	77.64	85
	VP7	**G41**	RVA/cow-tc/USA/cody I-801/xxxx/G8P[x]	U14999	73.12	80
	NSP1	**A38**	RVA/human-wt/JPN/HK14-5/2014/G1P[8]_A1	LC105017	58.90	79
	NSP2	**N27**	RVA/horse-tc/JPN/MK9/2019/G13P[18]_N9	LC528248	68.02	85
	NSP3	**T27**	RVA/human-wt/ARG/Arg4605/2006/G4P[6]_T7	KC412037	66.63	85
	NSP4	**E31**	RVA/bat-wt/CHN/YSSK5/2015/G3P[3]_E3	KX814962	61.29	85
	NSP5	**H27**	RVA/human-wt/SUR/2014735512/2013/G20P[28]	KX257411	71.20	91

a Closest strains were identified using BLASTn on megablast setting.

b Novel genotypes identified by the RCWG in this study were indicated in bold.

c Matthijnssens et al. (6)

To estimate potential reassortment events, the GCs of the isolated strains were compared to those of genetically and geographically related strains. RVA/Bat-wt/ZMB/ZFB14-126/2014/GxP[x] (ZFB14-126) was detected in *R. aegyptiacus* in Zambia, and it was shown this has the same E27 genotype as 16-06, with 87.7% nucleotide identity (Fig. 3A). ZFB14-126 has the I22 and T17 genotypes detected in Eidolon helvum-derived RVA from Zambia and Cameroon (25). In addition, RVA/Human-tc/KEN/B10/1987/G3P[2] (B10), originally detected in a human from Kenya, has the same I16 genotype as 16-06, with 87.4% nucleotide identity (Fig. 3A). B10 exhibits SA11-like GC in segments other than VP1, VP6, and NSP4. These data suggest that ZFB14-126 and B10 may have a reassortment history involving the ancestor of 16-06 and other strains, while complete the genotype constellation of ZFB14-126 remains to be determined.

Next, we performed phylogenetic analysis based on the isolated RVA strains with other bat-derived, rodent-derived, and type strains of each genotype. In VP7, 16-06 and other members of genotype G36 formed a single cluster with other bat RVAs assigned to genotype G25 (Fig. 3B). Similar tree topologies were observed in VP4, VP1, VP6, NSP1, NSP2, and NSP4 (Fig. 3C and Fig. S1). In contrast, 16-06 and other members of genotypes C20, M20, T22, and H22 segregated away from bat-specific genotypes and were phylogenetically closer to other mammalian RVAs in the VP2, VP3, NSP3, and NSP5 trees, respectively (Fig. S1). The observed phylogenetic incongruence could be due to (i) evidence of interspecies transmission and reassortment in the ancestor of 16-06 or (ii) potential lack of sequence data from undiscovered RVAs to fill gaps in the full phylogenetic tree. On the other hand, MpR12 formed distinct lineages to known RVAs and may have arisen from a closer common ancestor with bat RVA, but not with any known rodent RVAs in all segments except for NSP1 (Fig. 3B and C, and Fig. S1), highlighting the unique evolutionary history of MpR12.

### Glycan binding specificity of isolated RVAs.

RVA initiates infection via interaction between the VP8* domain of VP4 and cell-surface glycans, including sialic acids (SAs) or histo-blood group antigens (39). Liu et al. grouped RVAs into five genogroups (P[I] to P[V]) based on the amino acid sequence of VP8* and showed that the glycan-binding property restricts the host specificity of RVAs (40, 41). To investigate the glycan-binding properties of our isolated RVAs, we constructed phylogenetic trees based on the amino acid sequence of the VP8* region (Fig. S2A). In the tree, almost all P-genotypes were assigned into P[I] to P[V] genogroups. RVAs 16-06, 16-27, 18-12, and MpR12 were classified into the P[I] genogroup cluster with other known bat and rodent RVAs. The isolated RVAs diverged from the ancestor of the SA-dependent RVA group, consisting of the P[1], P[2], P[3], and P[7] genotypes (Fig. S2A). Accordingly, the conserved amino acid residues responsible for interaction with glycans were investigated based on the amino acid sequence alignment of VP8* (Fig. S2B). The residues interacting with SAs existed in VP8* of 16-06, 16-27, and 18-12, but not in that of MpR12, suggesting different SA-affinities of VP8* between the bat- and rodent-borne RVAs employed in this study. Amino acid sequence alignment of VP8 from MpR12 and other rodent RVAs showed that the conserved residues for interaction with sialic acid were not conserved in MpR12 (Fig. S2C).

**FIG 4:**
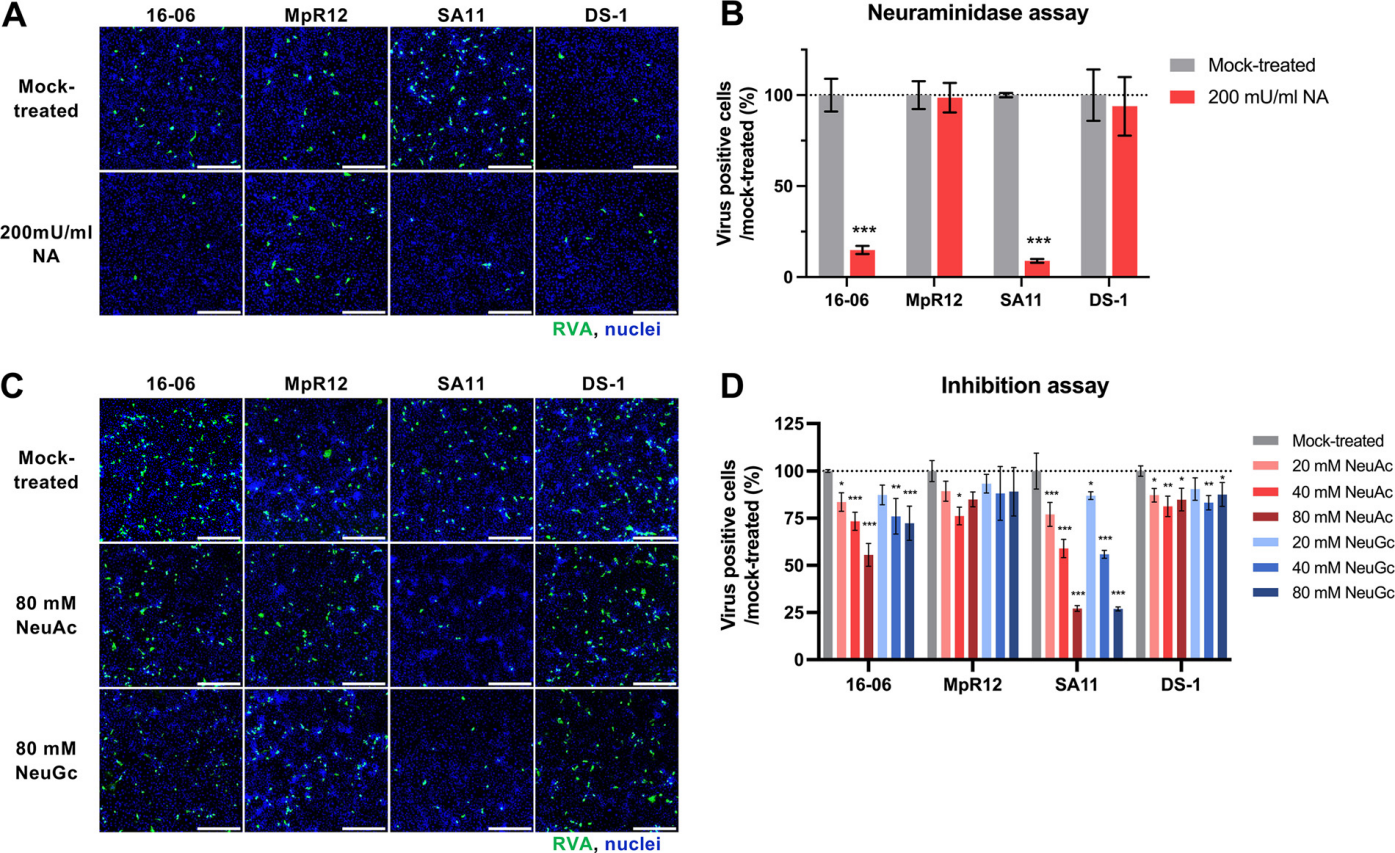
Involvement of sialic acid on the infectivity of the isolated RVAs. (A and B) MA104-T2T11D cells were pretreated with neuraminidase (NA) at 200 mU/mL or reaction buffer (mock-treated) and infected with 16-06 and MpR12. The SA11 and DS-1 strains were used as positive and negative controls, respectively. (C and D) MA104-T2T11D cells were infected with RVA pretreated with *N*-acetylneuraminic acid (NeuAc), *N*-glycolylneuraminic acid (NeuGc), or reaction buffer only (mock-treated). (A and C) Cells were stained with anti-RVA antibody (green) and Hoechst 33342 for nuclei (blue). Scale bars = 200 μm. The figures shown are representative images. (B and D) The number of infected cells with RVA is expressed as a percentage of the mock-treated control. Means ± SD of triplicate data from a representative experiment are shown in the graph. Statistical analysis was performed using multiple *t* tests with the Holm-Sidak method for the NA assay and a one-way ANOVA with Dunnett’s test for the SA inhibition test. ***, *P *<* *0.001; **, *P *<* *0.01; *, *P *<* *0.05.

To obtain direct evidence for the SA-dependency of the isolated RVAs, we investigated whether the enzymatic removal of SAs decreased the infectivity of the RVAs. Neuraminidase (NA) removes the terminal SAs from cell-surface glycans by cleaving the glycosidic bond of neuraminic acids and decreases SA-dependent infection of RVAs (42, 43). The SA-dependent SA11 strain in the P[I] genogroup was included in this assay as a positive control (41). Both Wa and DS-1 were clustered in the non-sialic acid dependent P[II] genogroup, but Wa binds to an internal sialic acid in the sugar chain and is sensitive to neuraminidase treatment (44). Thus, the DS-1 strain was selected as a negative control. Cell treatment with NA reduced the infectivity of 16-06 and SA11, but not that of MpR12 and DS-1 (Fig. 4A and B). This observation was consistent with the presence of amino acid sequences in VP8* (Fig. S2A and S2B). Next, we examined the neutralizing activity of SAs against RVAs using *N*-acetylneuraminic acid (NeuAc) and *N*-glycolylneuraminic acid (NeuGc). The monosaccharides NeuAc and NeuGc were individually preincubated with 16-06, MpR12, SA11, and DS-1 prior to infection. The infectivity of 16-06 and SA11 was reduced in a dose-dependent manner by NeuAc and NeuGc (Fig. 4C and D). Taken together, these data highlighted the difference in binding specificity to SAs between 16-06 and MpR12.

### Pathogenicity of isolated RVAs in suckling mice.

**FIG 5:**
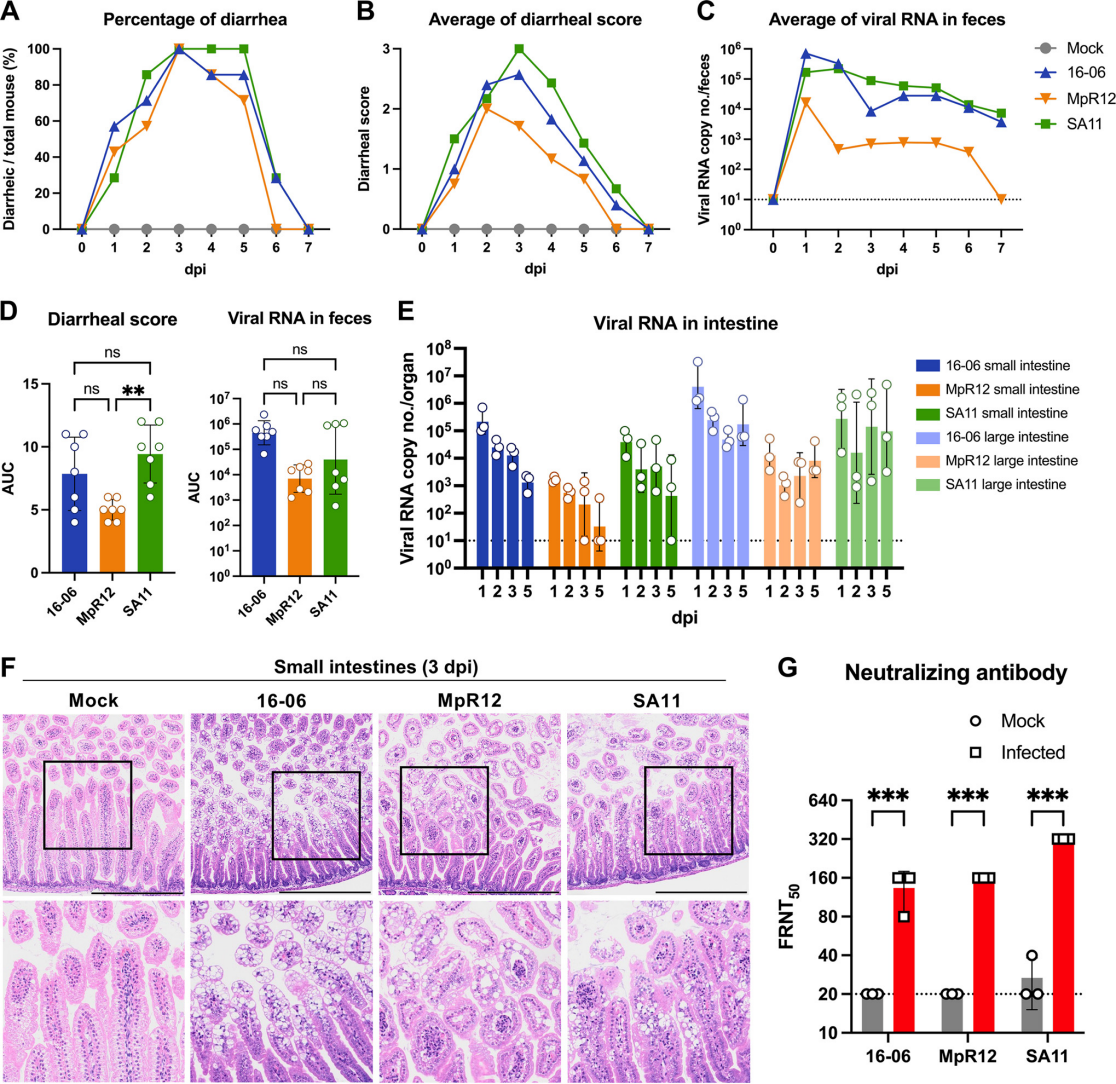
Pathogenicity of the isolated RVAs in suckling mice. Three-day-old BALB/c mice were orally inoculated with 1.0 × 10^5^ FFU of RVA strains 16-06, MpR12, SA11, or phosphate-buffered saline (mock) by gavage (*n *=* *7 in each group). (A) Incidence rate of diarrhea in each group was monitored from 0 to 7 days postinfection (dpi). (B) Fecal consistency in each group was scored according to the criteria described in Methods. (C) Average viral RNA copy numbers in feces from 0 to 7 dpi were calculated based on the results of qRT-PCR. Dashed line indicates detection limit of qRT-PCR. (D) Areas under the curves (AUCs) were calculated based on the diarrheal score and viral RNA copy number in feces of each mouse. (E) Viral RNA copy number of small and large intestines of infected mice at 1, 2, 3, and 5 dpi were determined using reverse transcription-quantitative PCR (qRT-PCR). (F) Infected sucking mice were euthanized at 3 dpi for histopathological examinations. Representative images of the small intestines of 16-06-, MpR12-, or SA11-infected mice and the control mice are shown. At 3 dpi, histopathological changes in the infected mice were characterized by vacuolization of the enterocytes in the villus tips. Hematoxylin and eosin staining (H&E). Scale bars = 500 μm. Areas in black squares are magnified in lower panels. (G) Neutralizing titers of mouse sera at 15 dpi were expressed as the dilution at which the number of viral foci was reduced by 50% compared to the no-serum control (FRNT_50_). Dashed line indicates detection limit of focus reduction neutralization test (FRNT). Means ± SD of each group from representative experiments are shown in the graph. Each dot represents one value from each mouse. Statistical analysis was performed using a one-way ANOVA with Tukey’s test for AUC analysis and multiple *t* tests with the Holm-Sidak method for the NA assay and the neutralizing antibody titers of suckling mice. ***, *P *<* *0.001; **, *P *<* *0.01; *, *P *<* *0.05.

To assess the infectivity and pathogenicity of the isolated bat and rodent RVAs, we used 3-day-old suckling mice in an experimental model. These mice were inoculated orally with RVA strains 16-06, MpR12, or SA11 (*n *=* *7 in each group). SA11, which causes diarrhea in suckling mice, was used as a control RVA (45, 46). The mock group was inoculated with phosphate-buffered saline (PBS) as a control. None of the suckling mice in each group died from 0 to 7 dpi. None of the mock-treated mice developed diarrhea during the observation period, while mice inoculated with RVA strains 16-06, MpR12, and SA11 developed diarrhea from 1 to 5 days postinfection (dpi) (Fig. 5A). SA11, 16-06, and MpR12 caused diarrhea in suckling mice with 100% morbidity (Fig. 5A). Diarrhea disease severity scores reached peak levels at 2 to 3 dpi in 16-06- and MpR12-infected mice, but the scores were lower than those of SA11-infected mice (Fig. 5B). Viral RNA copy numbers in the feces and intestines were quantified using a specific reverse transcription-quantitative PCR (qRT-PCR) assay for each strain with comparable detection sensitivity and amplification efficiency (Fig. S3). Viral RNA shedding peaked at 1 dpi and was continuously detected, with a gradual decrease, in the feces of 16-06-, MpR12-, and SA11-infected mice up to 7 dpi (Fig. 5C). We could not obtain feces from individual suckling mice after 7 dpi because they recovered from diarrhea. To estimate cumulative diarrheal severity and viral RNA shedding of infected mice, we calculated the area under the curve (AUC) based on the diarrheal score and viral RNA copy number in feces from each mouse. Mice inoculated with Wa and 16-06 displayed comparable cumulative diarrheal severity and viral RNA shedding, whereas MpR12 produced attenuated virulence in mice (Fig. 5D). Consistent with viral RNA in feces, the amount of viral RNA in the small intestines of infected mice peaked at 1 dpi and gradually decreased until 5 dpi, while the decrease in viral RNA signals was not clearly observed in the large intestine (Fig. 5E). Histopathological analysis revealed focal histopathological changes: vacuolization in the enterocytes lining most of the surface of the villi, with increased inflammatory cell infiltrates into the lamina propria in the small intestines of SA11-, 16-06-, or MpR12-infected mice (Fig. 5F) (47, 48). In particular, degeneration of surface epithelium was noted in the small intestine of SA11-infected mice. In contrast, these histopathological changes were not observed in animals in control group (Fig. 5F). To confirm RVA infection in suckling mice, we examined neutralizing antibody titers in mouse sera at 15 dpi using a focus reduction neutralization test (FRNT). All mice developed neutralizing antibodies against the inoculum strains (Fig. 5G and Fig. S4). In addition, we examined the transmissibility of the isolated strains from infected suckling mice to uninfected littermates. Compared with oral administration, cohabitation infection caused mild diarrhea and RNA shedding in suckling mice (Fig. S5). These data indicated that oral administration of 16-06 and MpR12 caused diarrheal disease and viral shedding in suckling mice, and that MpR12 has attenuated growth and pathogenicity compared with 16-06 and SA11 in mice.

### Infection and growth of isolated RVAs in a human small intestinal epithelial model.

**FIG 6:**
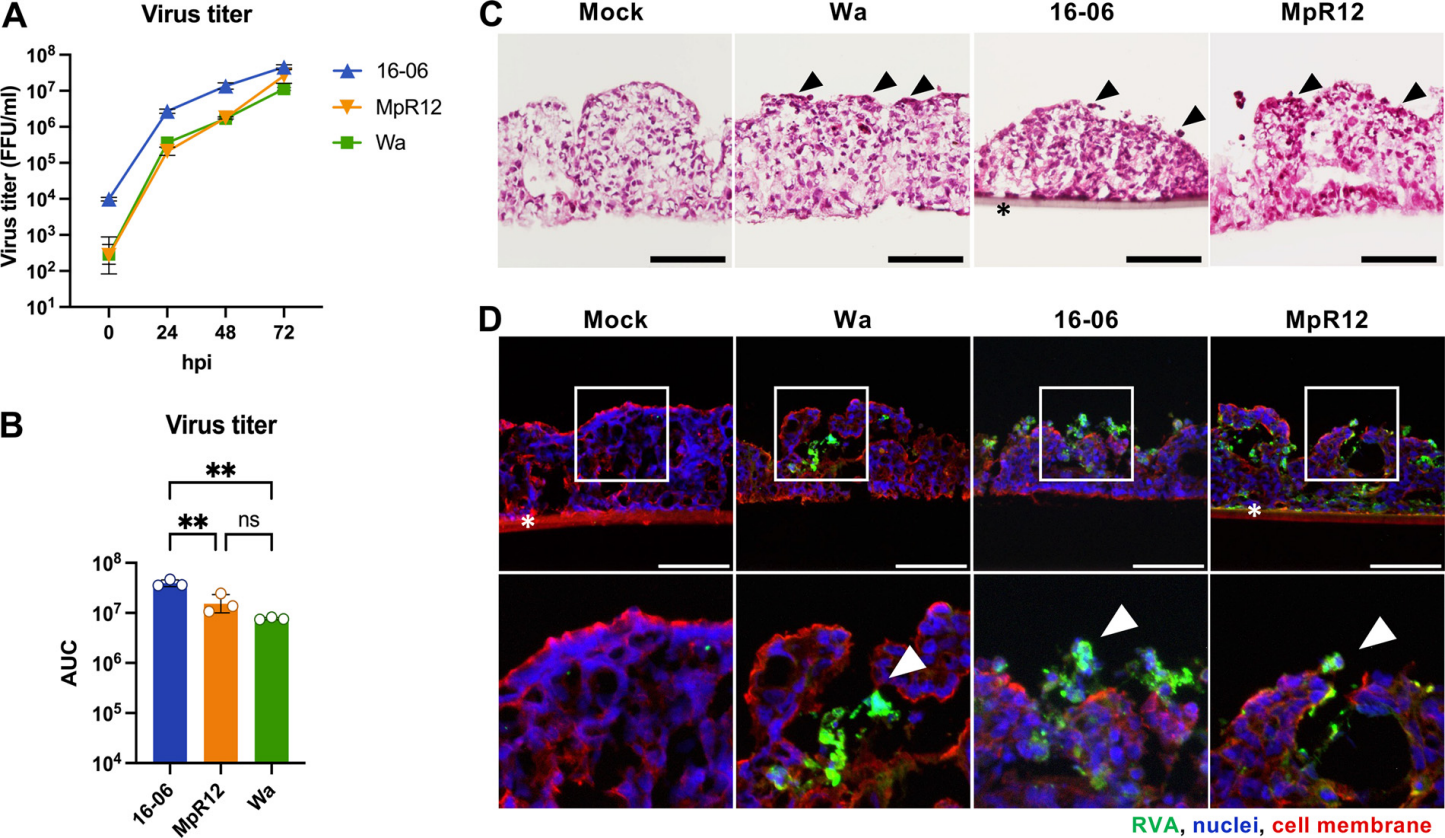
Infectivity of the isolated RVAs in a human small intestinal epithelial *ex vivo* model, SMI-100. (A) Growth kinetics of 16-06, MpR12, and Wa in SMI-100. SMI-100 was inoculated with 1.0 × 10^5^ FFU of 16-06, MpR12, or Wa. Supernatants were collected at the indicated time points (hpi), and virus titers were measured by a focus assay. Means ± SD of triplicate data from a representative experiment are shown in the graph. (B) AUCs based on the viral titers in the culture supernatants of each SMI-100-well infected with 16-06, MpR12, or Wa. Means ± SD of triplicate data from a representative experiment are shown in the graph. Each dot represents one AUC value from each SMI-100 well. Statistical analysis was performed using a one-way ANOVA with Tukey’s test. ***, *P *<* *0.001; **, *P *<* *0.01; *, *P *<* *0.05. (C) Histopathological images of vertical sections of SMI-100 at 3 dpi with H&E staining. Black arrowheads indicate acidophilic dead cells with fragmented nuclei. Scale bars = 100 μm. (D) Vertical sections of SMI-100 at 3 dpi were stained for RVA (green), nuclei (blue), and cell membrane using wheat germ agglutinin (red). Scale bars = 100 μm. Areas in white squares are magnified in lower panels. White arrowheads indicate exfoliated infected cells. Asterisks indicate the mesh membranes which support the epithelium. The figures shown are representative images.

We next examined the infectivity of 16-06 and MpR12 in an *ex vivo* model of human small intestinal epithelium: the EpiIntestinal Small Intestine Tissue Model (SMI-100). SMI-100 is 3D-reconstructed from human primary intestinal epithelial cells and exhibits a tissue structure similar to that of small intestine tissues (49). We inoculated 16-06, MpR12, and the human RVA Wa strain on the apical area of SMI-100 to mimic infection from the luminal side of the intestine. The Wa strain was selected as a positive control because it has been employed in other studies of *ex vivo* RVA infection models (50, 51). The titers of 16-06 and MpR12 in culture supernatants increased in a time-dependent manner, reaching over 10^7^ focus forming units (FFU)/mL at 72 hpi (Fig. 6A). The growth curve of 16-06 was higher than that of Wa, whereas MpR12 exhibited a growth efficiency comparable to Wa. The AUC of viral titers in the culture supernatants of each SMI-100 culture insert shows that 16-06 produced progeny virus at a significantly higher titer than MpR12 and Wa (Fig. 6B). Histopathological analysis showed acidophilic dead cells containing fragmented nuclei on the apical surface of SMI-100 infected with 16-06, MpR12, and Wa (Fig. 6C). Immunohistochemistry identified RVA antigen signals in the enterocytes located at the villus tips and detached cells from the apical surface at 3 dpi with 16-06, MpR12, and Wa (Fig. 6D). These data demonstrated the ability of 16-06 and MpR12 to infect and replicate in the human intestinal epithelium.

## DISCUSSION

Recent advances in virus genome detection methods and RVA genotype classification have revealed the great diversity of animal RVAs and led to the identification of multiple new genotypes. However, most RVAs belonging to the new genotypes have been identified from genomic RNA, not from isolated infectious viruses, and have not been subjected to subsequent virological characterization (18, 21–24, 26, 28, 30). There are two difficulties in the detection and isolation of RVA from wild animals. The first is that the large sequence diversity of the RVA genome hampers RT-PCR with broadly reactive consensus primers. A viral metagenomic approach is one practical strategy to identify diverse RVA strains with low sequence similarities to known RVA strains, but this method is not suitable for screening animal RVAs with large number of specimens considering the low prevalence rate of RVA in wild animals (<10%) (28, 30). The second issue is the protease-dependent infectivity of RVA. RVA-inoculated cells are usually maintained in a serum-free medium supplemented with trypsin to cleave and activate viral spike protein VP4, which is essential for RVA entry (37). However, some inocula, such as feces, exhibit toxicity to cells under serum-free conditions. To overcome this limitation, we employed virus isolation-based RVA screening using MA104-T2T11D cells, successfully isolating three strains of bat RVA and one strain of rodent RVA (Fig. 1). The RVA isolation rate of *R. aegyptiacus* was 0% to 9.1%, and that of *M. natalensis* was 3.6% (Table 1), which is consistent with the RVA genome positivity rate in wild animals reported in previous studies (28, 30). Therefore, virus isolation-based RVA screening offers an alternative approach to conventional RT-PCR for large-scale and sensitive RVA detection from animal and human specimens, especially atypical RVAs with previously unrecognized genotypes.

Bat RVA 16-06, isolated in this study, has the same GC (G36-P[51]-I16-R22-C20-M20-A31-N22-T22-E27-H22) as bat RVA strain BATp39 (Table 2). According to GenBank, the genome sequence of BATp39 was detected in *R. aegyptiacus* in Kenya in 2015; however, detailed information on the BATp39 strain is currently lacking. The identification of bat RVAs with the same GC from *R. aegyptiacus* in Kenya and Zambia indicates that this GC may be widespread in East Africa. Our GC analysis also revealed evidence of genome reassortment between ancestors of bat-derived RVAs and the atypical human RVA strain B10 (Fig. 3A). Notably, glycan-binding analysis suggests that 16-06 as well as SA11 recognize both human and animal-type SA, NeuAc and NeuGc (Fig. 4) (52). It has been reported that the property of glycan-binding is involved in the host specificity of RVA (40, 41). Simsek et al. have identified SA11-related RVA in Gabonese bats, suggesting a multi-species host range of SA11-related RVAs (28). The similarities in sugar chain utilization of 16-06 and SA11 raise the question whether 16-06 and relative RVAs are bat-specific or cross-species transmissible viruses.

To date, RVAs have been detected in wild rodents (*Mus* sp., *Rattus* sp., *Niviventer* sp., and *Apodemus* sp. in the family Muridae) in Germany, China, and the United States (30–33). Due to the limited number of rodent RVAs identified, little is known about the genetic diversity and evolution of rodent viruses. MpR12 is the first rodent RVA detected from Natal multimammate mouse (*M. natalensis*). While *Mastomys* sp. is phylogenetically close to *Mus* sp. and *Niviventer* sp. (53), our phylogenetic analysis revealed that MpR12 was more related to bat RVA rather than other rodent RVAs (Fig. 3B and C and Fig. S1). In addition, all segments of MpR12 were distinct from any other RVAs and were assigned to novel genotypes. The origin and evolution of MpR12 remain to be elucidated.

The potential infectivity of some bat and rodent RVAs to humans has been speculated based on the detection of reassortment between these RVAs and human RVAs, but the zoonotic potential of bat and rodent RVAs needs to be further investigated (18, 21–24, 26, 28, 30). Recently, human intestinal enteroids have been used as cellularly diverse and physiologically relevant models for human RVA infection (50). Here, we used a 3D-reconstructed human small intestinal epithelium, SMI-100, as an alternative *ex vivo* model to assess the infectivity of the isolated RVA strains to the human gut. SMI-100 was susceptible to infection by 16-06 and MpR12 and permitted growth at levels similar to that of human RVA (Fig. 6). Few cell lines, including monkey kidney derived-MA104 and CV-1, have the capacity to propagate RVA infection. To the best of our knowledge, this is the first study to use SMI-100 for RVA infection, and it appears to be a relevant and reliable tool to study multiple aspects of RVA infection in the human gut.

One limitation of this study is the lack of direct evidence for the zoonotic potential of the isolated RVA strains. Some animal RVA can actively propagate even in human organoids but does not cause disease in humans because the host range of RVA is affected by a wide range of factors, including host age and sex, the accessibility of susceptible cells, and immune response (42). RVA surveillance of human in Zambia have not detected RVA with the genotypes reported in this study, as human RVAs with typical genotypes are the main targets of the surveillance (54–57). To clarify the zoonotic transmission of RVA between humans and wild animals, further surveillance should be conducted on human clinical samples in Zambia.

In conclusion, we have isolated novel bat and rodent RVA strains by virus isolation-based RVA screening. Furthermore, whole-genome analysis, glycan utilization analysis, experimental inoculation in suckling mice, and infectivity in a human small intestinal epithelial model enabled characterization of the unique virological properties of the isolated RVAs and have highlighted their zoonotic potential.

## MATERIALS AND METHODS

### Sample collection.

A total of 325 fruit bat, 48 rodent, and 24 shrew archived samples collected in Lusaka, Shimabala, and Mpulungu in Zambia from 2012 to 2018 (Fig. 1A and Table 1) were used in this study. Bats were captured with harp traps and rodents and shrews were captured with Sherman traps and cage traps. Captured animals were euthanized with diethyl ether, and the contents of the large intestines were collected and kept at −80°C. Bats were morphologically classified and rodents and shrews were classified by nucleotide sequence analysis of the mitochondrial *cytochrome b* gene, as described previously (58). The biological samples were collected under the approval of the Zambia Wildlife Authority, now the Department of National Parks and Wildlife, Ministry of Tourism and Arts, Zambia (DNPW8/27/1). The University of Zambia Biomedical Research Ethics Committee gave ethical approval for this study (REF. NO. 1382-2020).

### Cells and viruses.

Rhesus monkey kidney MA104 cells were maintained in Eagle’s minimum essential medium (MEM; Nissui, Tokyo, Japan) supplemented with 10% fetal bovine serum (FBS), 10% tryptose phosphate broth (TPB), 100 U/mL penicillin, and 100 μg/mL streptomycin (PS). MA104-T2T11D cells were generated using the lentiviral vector system as described previously (34). Simian RVA SA11 (VR-1565; ATCC, Manassas, VA), human RVA Wa (VR-2018; ATCC) and human RVA DS-1 (VR-2550; ATCC), and bovine RVA Azuk-1 strains were propagated in MA104 cells in serum-free MEM containing 10% TPB, PS, and trypsin (0.5 μg/mL) under rotary conditions. Virus titers were determined by a focus assay as described previously (34).

### Virus isolation workflow.

Fecal suspensions or intestinal homogenates of bats, rodents, and shrews were centrifuged at 3,000 × *g* for 5 min, and supernatants were filtrated through Vivaclear Mini Centrifugal 0.8-μm filters (Sartorius, Goettingen, Germany). Flowthrough was inoculated to MA104-T2T11D cells with 2 mL isolation medium (MEM supplemented with 10% FBS, 10% TPB, PS, 25 μg/mL gentamicin, 1% antibiotic-antimycotic solution [Wako, Osaka, Japan] and 15 mM HEPES) in 15-mL tissue culture tubes (TPP, Trasadingen, Switzerland). Cells with the inoculum were rotated at 0.3 rpm/min for 7 days. After a single freeze-thaw cycle, part of the cell suspension (P1 culture) was blindly passaged in fresh MA104-T2T11D cells. A portion of passaged culture (P2 culture) was then pooled and ultracentrifuged at 110,880 × *g* for 2 h with a 20% sucrose cushion, and then pellets were subjected to nucleic acid extraction and NGS analysis as described below. The remaining P2 culture was used for RT-PCR screening for RVAs identified in NGS analysis. For RT-PCR, total RNA of P2 culture supernatants was extracted using a High Pure Viral Nucleic Acid kit (Roche, Basel, Switzerland) and subjected to RT-PCR analysis using PrimeScript One Step RT-PCR kit version 2 (TaKaRa Bio, Shiga, Japan). The primer and probe sequences are listed in Table S3. The identified culture supernatants were further passaged in MA104-T2T11D to prepare working virus stocks for subsequent experiments.

### Nested RT-PCR screening in bat and rodent feces.

Total RNA was extracted from fecal suspensions or intestinal homogenates of bats, rodents, and shrews using a High Pure Viral Nucleic Acid kit. First, RT-PCR was performed using a SuperScript IV One-Step RT-PCR System (Thermo Fisher Scientific, Waltham, MA) with the following thermal cycling conditions: 50°C for 10 min, 98°C for 2 min, 40 cycles of 98°C for 10 s, 55°C for 10 s, and 72°C for 30 s, and finally 72°C for 5 min. The initial PCR products were subjected to a second PCR using Tks Gflex DNA polymerase (TaKaRa Bio) with the following thermal cycling conditions: 98°C for 2 min, 40 cycles of 98°C for 10 s, 55°C for 15 s, and 68°C for 30 s, and finally 68°C for 5 min. The primer sequences are listed in Table S3.

### Transmission electron microscopy.

For negative-stain electron microscopy, RVA virions in the culture were pelleted by ultracentrifugation for 2 h at 110,880 × *g* with a 20% sucrose cushion and resuspended in PBS. The concentrated RVAs were fixed with 4% paraformaldehyde, deposited on a nickel grid coated with polyvinyl formal (Nissin EM, Tokyo, Japan) and stained with 2% phosphotungstic acid (pH 5.8). Samples were observed under a transmission electron microscope (H-7650; Hitachi High-Technologies, Tokyo, Japan).

### Indirect immunofluorescence assay.

MA104-T2T11D cells infected with RVA were fixed with 3.7% buffered-formaldehyde and permeabilized with ice-cold methanol. Subsequently, cells were incubated for 1 h with anti-RVA polyclonal antibody (AB1129; Merck, Burlington, MA) as the primary antibody at a 1:500 dilution in PBS with 25% Block Ace (KAC, Kyoto, Japan). After three washes with PBS, secondary staining was performed with Alexa Fluor 488-conjugated anti-goat IgG antibody (A-11055; Invitrogen: Thermo Fisher Scientific, 1:1,000) and 10 μg/mL Hoechst 33342 (Invitrogen) for 1h. Fluorescence images were captured using a fluorescence microscope (IX73; Olympus, Tokyo, Japan).

### Whole-genome sequencing.

Viral RNA was extracted from working stocks of 16-06, 16-27, 18-12, and MpR12 using a High Pure Viral Nucleic Acid kit, reverse-transcribed into double-stranded cDNA using a PrimeScript Double Strand cDNA Synthesis kit (TaKaRa Bio), and then subjected to sequence library construction using a Nextera XT DNA Library Preparation kit (Illumina, San Diego, CA). The 300-bp paired-end sequencing was performed on an Illumina MiSeq sequencer (Illumina). Sequence reads were trimmed and assembled into contigs by *de novo* assembly using CLC Genomics Workbench 21 (Qiagen, Hilden, Germany). The obtained contigs were analyzed by the BLASTn program (National Center for Biotechnology Information, Bethesda, MD). The 5′- and 3′-terminal sequences of each genome segment were determined using a SMARTer RACE 5′/3′ kit (TaKaRa Bio) with the segment-specific primers listed in Table S3. The GCs of isolated strains were assigned based on whole-genome sequences in the Rotavirus A Genotype Determination tool in ViPR (https://www.viprbrc.org/brc/home.spg?decorator=vipr) provided by the RCWG (38).

### Phylogenetic analysis.

The genome sequences of isolated RVAs were aligned with reference RVA sequences from GenBank using the MUSCLE algorithm with the default parameters in CLC Genomics Workbench 21. Information on reference strains is listed in Table S4. Maximum-likelihood (ML) trees were constructed using models of general time-reversible with gamma rate categories and invariant sites (GTR+G+I) for full-length ORFs of VP1, VP2, VP3, VP4, VP7, NSP1, and NSP2; and general time-reversible with gamma rate categories (GTR+G) for VP6, NSP3, NSP4, and NSP5 as the best fit models based on minimum Akaike information criterion (AIC), with bootstrap values of 1,000 replicates in the MEGA X software (59). The phylogenetic trees were visualized and annotated in Interactive Tree Of Life (iTOL) version 6.5.2 (60).

### Neuraminidase assays.

NA assays were carried out as described previously with slight modifications (42). Briefly, confluent monolayer MA104-T2T11D cells were pretreated with 200 mU/mL NA from Vibrio cholerae (Sigma-Aldrich, St. Louis, MO) in MEM with 25 mM HEPES and 9 mM CaCl_2_ (pH 6.0) at 37°C for 1 h. Either mock- or NA-treated cells were infected with each RVA strain at an MOI of 0.1 (strains SA11 and 16-06) or 1 (strains DS-1 and MpR12) at 37°C for 1 h. After the inoculum was removed, cells were cultured for 16 h in the overlay medium (MEM supplemented with 2% FBS and 0.5% methylcellulose). RVA-infected cells and cell nuclei were stained by anti-RVA antibody and Hoechst 33342 as described above, respectively, and counted using an IN Cell Analyzer 2500 (GE Healthcare, Waukesha, WI).

### Sialic acid inhibition assays.

SA inhibition assays were carried out as described previously with slight modifications (42). Briefly, viruses were preincubated with various concentrations of NeuAc (Wako) or NeuGc (Cayman Chemical, Ann Arbor, MI) in 2% FBS MEM with 25 mM HEPES (pH 7.6) for 2 h. The mixture was then added to a confluent monolayer of MA104-T2T11D cells at an MOI of 0.1 (strains SA11 and 16-06) or 1 (strains DS-1 and MpR12) at 37°C for 1 h. After removing the inoculum, cells were cultured for 16 h in the overlay medium (MEM supplemented with 2% FBS and 0.5% methylcellulose). RVA-infected cells and cell nuclei were counted as described above.

### Experimental infection in suckling mice.

All animal experiments were performed following the Regulations on Animal Experimentation of Hokkaido University, and the protocol was approved by the Institutional Animal Care and Use Committee of Hokkaido University (approval no. 20-0026). Litters of 3-day-old BALB/c mice were inoculated orally with 1.0 × 10^5^ FFU of RVA strain SA11, 16-06, or MpR12 by gavage (*n *=* *7 in each group). Control mice were treated with PBS as a mock-infected group (*n *=* *7). The conditions of feces were monitored by palpation of the abdomen every day from 0 to 7 dpi. The state of the stool was classified into four categories based on the color, texture, and amount of feces according to the criteria used in a previous study: 0, normal feces; 1, exceptionally loose feces; 2, loose yellow feces; and 3, liquid feces (47). Stools with a score of ≥1 were considered diarrheal stools. Feces, small intestines, and large intestines were collected and suspended in PBS following RNA extraction using TRIzol LS (Thermo Fisher Scientific) and the Direct-zol RNA miniprep kit (Zymo Research, Irvine, CA). Extracted RNAs were subjected to qRT-PCR analysis using a Thunderbird probe one-step qRT-PCR kit (TOYOBO, Osaka, Japan). The primer and probe sequences are listed in Table S3 (61). Sera for FRNT were collected from suckling mice (*n *=* *3 in each group) at 15 dpi.

### Focus reduction neutralization test.

After inactivation at 56°C for 30 min, mouse sera were 2-fold serially diluted and then incubated 1:1 with each virus (200 FFU/well) at 37°C for 1 h. The mixtures of serum and virus were then inoculated to MA104-T2T11D cells and cultured for 18 h. The foci were immunostained as described above and counted for neutralizing activity, expressed as the dilution factor at which the number of viral foci was reduced by 50% compared to the no-serum control (FRNT_50_).

### RVA-infection in a human small intestinal epithelial model.

Human EpiIntestinal Small Intestine Tissue Models (SMI-100; MatTek Life Science, Ashland, MA) were maintained with SMI-100 maintenance medium (MatTek) according to the manufacturer’s instructions. Subsequently, the apical surfaces of SMI-100 were infected with 1.0 × 10^5^ FFU of RVA in 100 μL of SMI-100 maintenance medium. After 6 h of incubation, the apical areas of SMI-100 were washed three times with PBS and fed with 100 μL of FBS-free MEM supplemented with 250 μg/mL trypsin in the apical areas. Progeny RVA in the culture supernatants in the apical areas were collected at each time point and titrated by a focus assay. At 72 hpi, RVA-infected SMI-100 were fixed with 3.7% buffered-formaldehyde and subjected to histopathology and immunohistochemistry.

### Histopathology and immunohistochemistry.

Mouse tissue samples were immersed in 3.7% buffered-formaldehyde and fixed. Next, the fixed tissue specimens were embedded in paraffin. Tissue sections (3 μm) were cut and mounted onto glass slides for either standard hematoxylin and eosin (H&E) staining. Histopathological images were acquired with a slide scanner (SLIDEVIEW VS200, Olympus, Tokyo, Japan). Fixed SMI-100 was embedded in the Tissue-Tek O.C.T. compound (Sakura Finetek Japan, Tokyo, Japan) and frozen at −80°C. Frozen tissue blocks were cut into 6-μm thick sections and mounted on CREST coat slides (Matsunami, Osaka, Japan). For histopathological analysis, slides were stained with H&E. For immunohistochemistry, slides were permeabilized with ice-cold ethanol, washed with PBS, and stained with anti-RVA polyclonal antibody (AB1129; Merck) as the primary antibody in PBS with 25% Block Ace. After three washes with PBS, secondary staining was performed with Alexa Fluor 488-conjugated anti-goat IgG antibody (A-11055, 1:1,000), 10 μg/mL Hoechst 33342, and wheat germ agglutinin, Alexa Fluor 594 Conjugate (W11262; Invitrogen, 1:100). Fluorescence images were captured using an IX73 fluorescence microscope.

### Statistical analysis.

Data were represented as the mean ± standard deviation (SD). AUC was calculated using Prism 9 (GraphPad Software, San Diego, CA). Statistical analysis was performed using Student’s *t* test for the growth kinetics assay, multiple *t* tests with the Holm-Sidak method for the NA assay and the neutralizing antibody titers of suckling mice, one-way analysis of variance (ANOVA) with Dunnett’s test for the SA inhibition test, and one-way ANOVA with Turkey’s test for the AUC analysis using Prism 9 (GraphPad Software).

### Data availability.

The GenBank/EMBL/DDBJ accession numbers for the viral sequences reported in this paper are as follows: LC704642 to LC704652 (RVA/Bat-tc/ZMB/16-06/2016/G36P[51]), LC704653 to LC704663 (RVA/Bat-tc/ZMB/16-27/2016/G36P[51]), LC704664 to LC704674 (RVA/Bat-tc/ZMB/18-12/2018/G36P[51]), and LC638698 to LC638708 (RVA/MultimammateMouse-tc/ZMB/MpR12/2012/G41P[57]).
