# Supplementary material for: Isolation and Characterization of Distinct Rotavirus A in Bat and Rodent Hosts
Source: J Virol. 2023 Jan 12;97(1):e01455-22. doi: 10.1128/jvi.01455-22 (PMC9888233; doi:10.1128/jvi.01455-22)
Supplement: Supplemental file 2 — Fig. S1 to S5. Download jvi.01455-22-s0002.pdf, PDF file, 5.7 MB [file jvi.01455-22-s0002.pdf]

Fig. S1

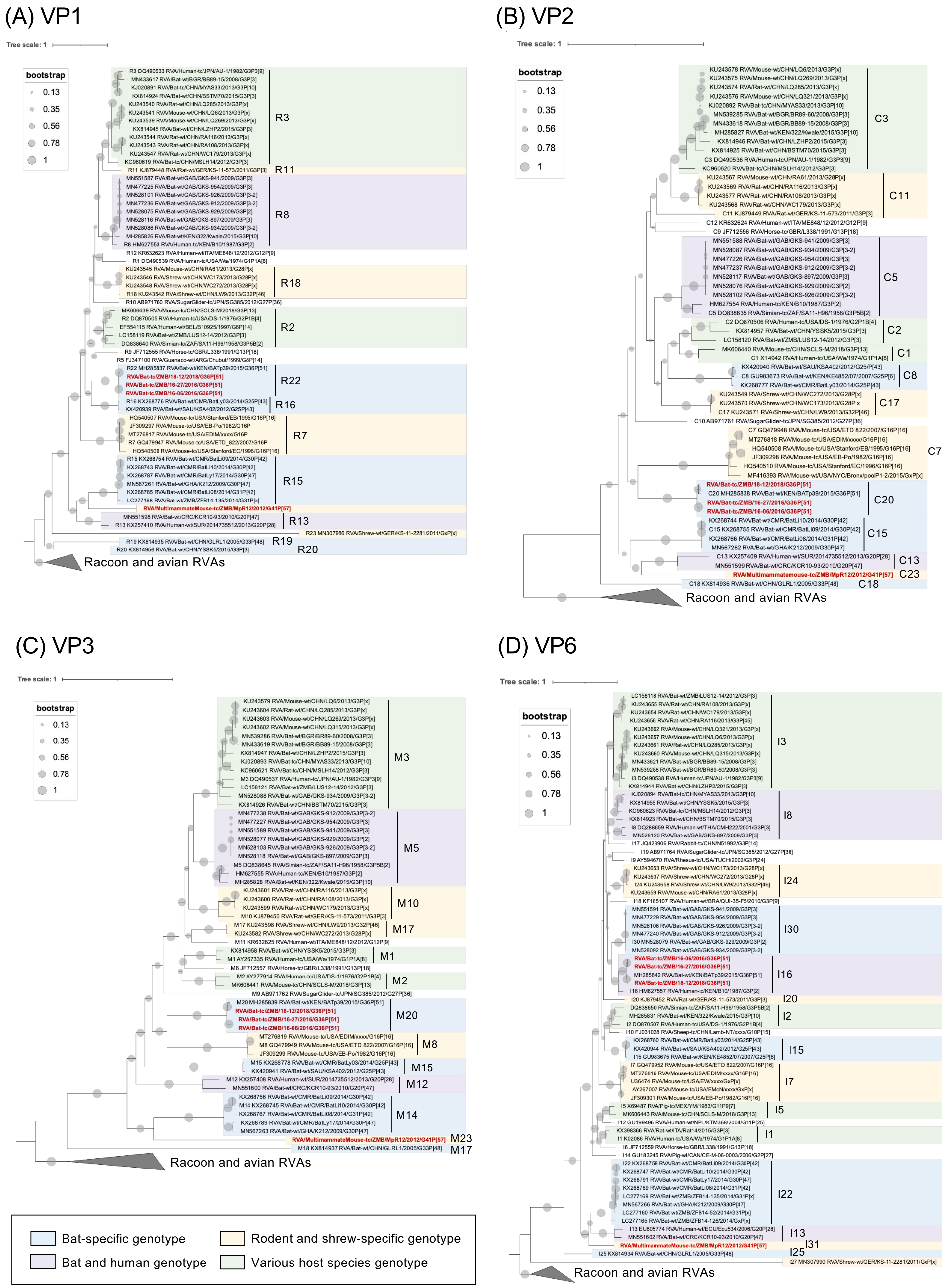
(A) VP1
Tree scale: 1
bootstrap
0.13
0.35
0.56
0.78
1
R3
R11
R8
R18
R2
R22
R16
R7
R15
R13
R19
R20
Racoon and avian RVAs
(B) VP2
Tree scale: 1
bootstrap
0.13
0.35
0.56
0.78
1
C3
C11
C5
C2
C1
C8
C17
C7
C20
C15
C13
C23
C18
Racoon and avian RVAs
(C) VP3
Tree scale: 1
bootstrap
0.13
0.35
0.56
0.78
1
M3
M5
M10
M17
M1
M2
M20
M8
M15
M12
M14
M23
M17
Racoon and avian RVAs
(D) VP6
Tree scale: 1
bootstrap
0.13
0.35
0.56
0.78
1
I3
I8
I24
I30
I16
I20
I2
I15
I7
I5
I1
I22
I13
I31
I25
Racoon and avian RVAs
Bat-specific genotype
Rodent and shrew-specific genotype
Bat and human genotype
Various host species genotype

Fig. S1

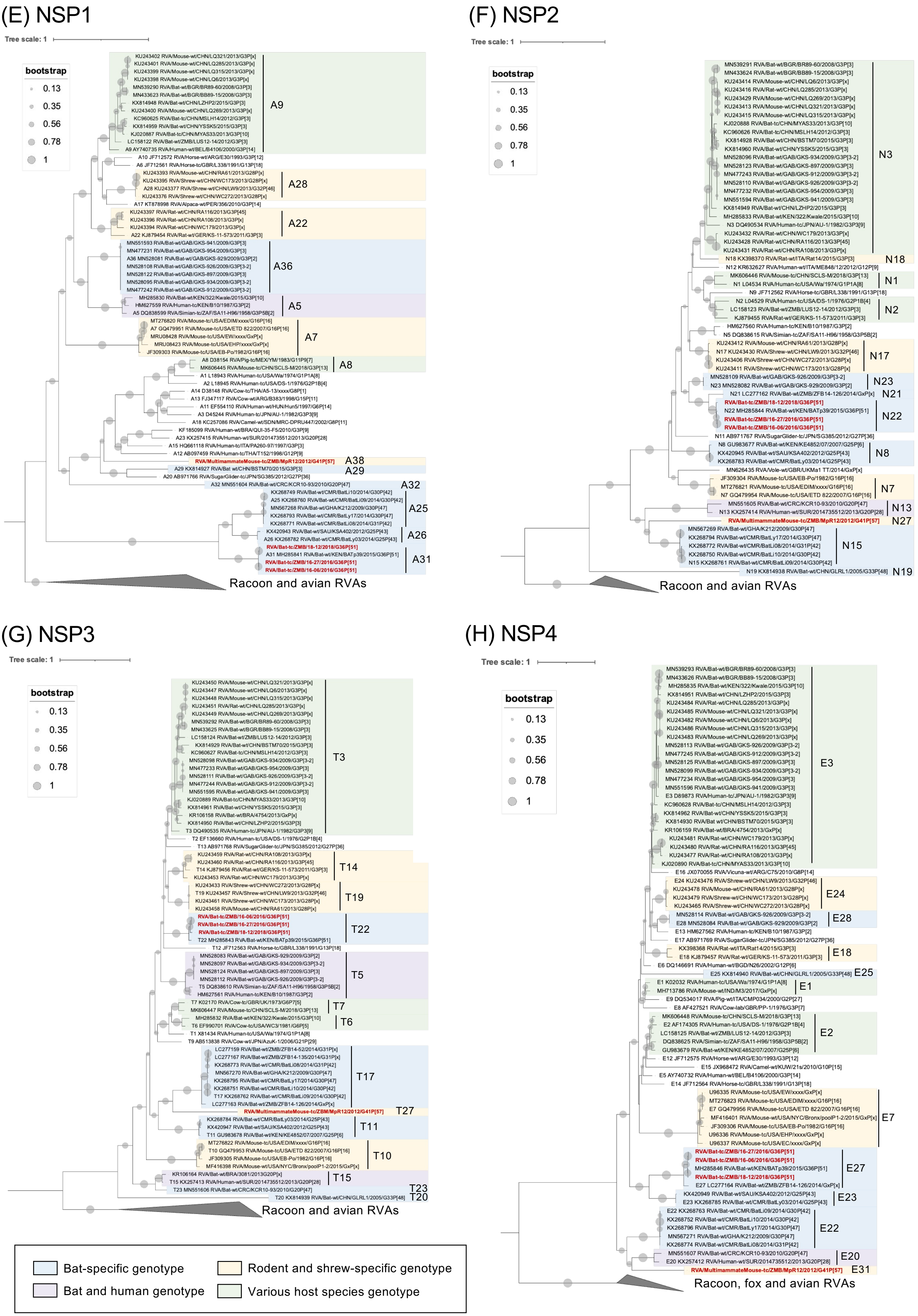

(E) NSP1
Tree scale: 1
bootstrap
0.13
0.35
0.56
0.78
1
A9
A28
A22
A36
A5
A7
A8
A38
A29
A32
A25
A26
A31
Racoon and avian RVAs
(F) NSP2
Tree scale: 1
bootstrap
0.13
0.35
0.56
0.78
1
N3
N18
N1
N2
N17
N23
N21
N22
N8
N7
N13
N27
N15
N19
Racoon and avian RVAs
(G) NSP3
Tree scale: 1
bootstrap
0.13
0.35
0.56
0.78
1
T3
T14
T19
T22
T5
T7
T6
T17
T27
T11
T10
T15
T23
T20
Racoon and avian RVAs
(H) NSP4
Tree scale: 1
bootstrap
0.13
0.35
0.56
0.78
1
E3
E24
E28
E18
E25
E1
E2
E7
E27
E23
E22
E20
E31
Racoon, fox and avian RVAs
Bat-specific genotype
Rodent and shrew-specific genotype
Bat and human genotype
Various host species genotype

Fig. S1

(I) NSP5

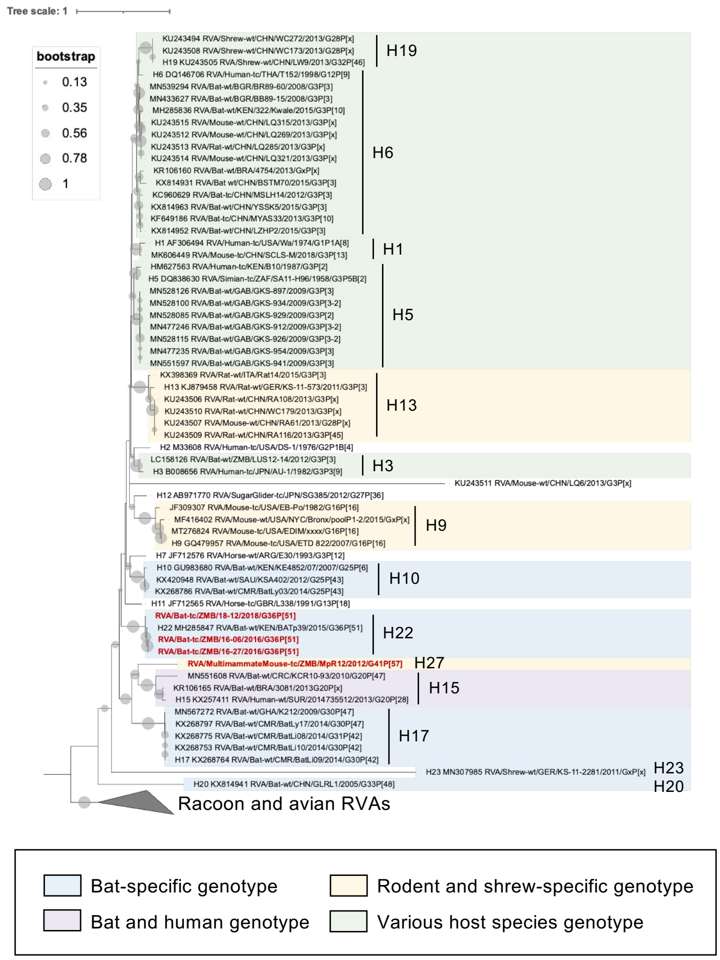

Fig. S1. Maximum-likelihood trees of VP1 (A), VP2 (B), VP3 (C), VP6 (D), NSP1 (E), NSP2 (F), NSP3 (G), NSP4 (H), and NSP5 (I) genes based on the sequence of the isolated RVAs, bat-derived RVAs, rodent-derived RVAs, and type strains of each genotype. Phylogenetic trees were constructed by the Maximum-Likelihood method using models of GTR+G+I for VP1, VP2, VP3, NSP1, and NSP2 and GTR+G for VP6, NSP3, NSP4, and NSP5 with bootstrap values of 1,000 replicates. Avian, raccoon, and fox RVAs were included as the outer group. The isolated RVAs are indicated in red color. Bat-specific genotypes and rodent and shrew-specific genotypes are highlighted in blue and yellow, respectively. The genotypes include bat-derived and non-typical human RVAs are colored in purple. The genotypes consisting of RVAs from multiple animal species are highlighted in green.

Fig. S2

A

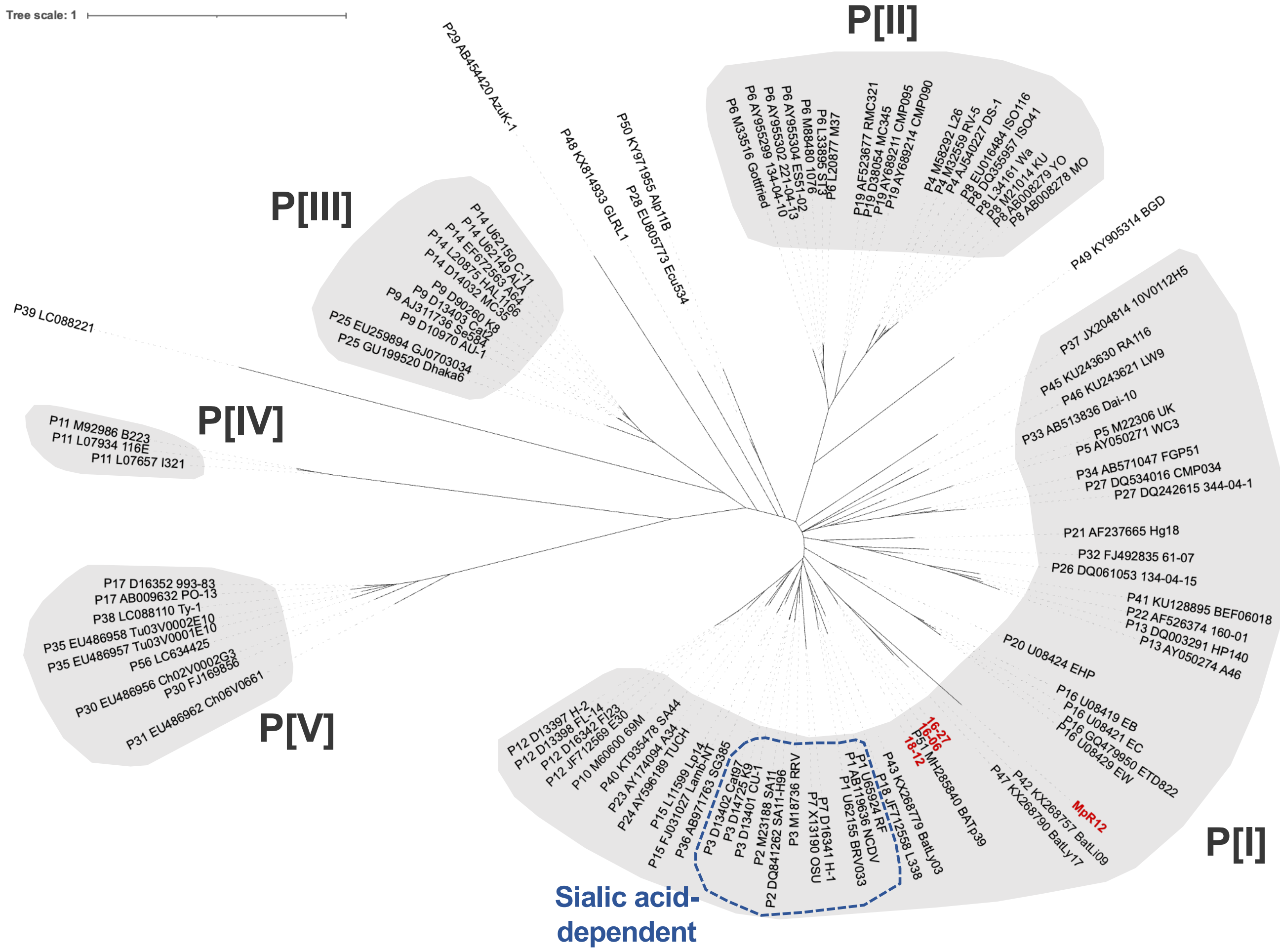

B

	Strain	100		150		170 190		215
	MpR12	NKW	···	LIKTSADGKYTEVAP	···	IMKYSNAIYKFMGSTPNTTLGFHSM	···	SRENQN
	16-06	NRW	···	VIKTTVNGTYTQYAP	···	VMKHDGKIYTYNGSTPNATTGYYST	···	PRSNEA
	16-27	NRW	···	VIKTTVNGTYTQYAP	···	VMKHDGKIYTYNGSTPNATTGYYST	···	PRSNEA
	18-12	NRW	···	VIKTTVSGTYTQYAP	···	VMKHDGKIYTYNGSTPNATTGYYST	···	PRSNEA
P[I]	P51_BATp39	NRW	···	VIKTTVNGTYTQYAP	···	VMKHDGKIYTYNGSTPNATTGYYST	···	PRSNEA
	P1_NCDV	NRW	···	LSKQTQDGNYSQHGS	···	VMKHGGKIYTYNGETPNATTGYYST	···	PLAQEA
	P2_SA11	DRW	···	VVKTTANGSIGQYGS	···	VMKHNEKLYTYEGQTPNARTGHYST	···	PRSEES
	P3_RRV	DRW	···	VVKTTQNGSYSQYGP	···	VMKHNGKIYTYNGETPNVTTKYYST	···	PREEES
	P7_H-1	DRW	···	VSKTTLTGNYTQHGP	···	IMRFSGRIYTYNGTTPNATTGYYST	···	PRNQEE
	P10_69M	NRW	···	LMKTTSSGTYTQHSP	···	IMKHGGQLWTYNGETPNAITNGYPT	···	PRSQES
	P12_H-2	NRW	···	LIKTTLSGNFTLYST	···	IMKHGGQLWVYNGETQTLLLQDYVT	···	PRSQES
P[II]	P19_MC345	DVW	···	MFRNNSNAEFQHKRT	···	ILKHGGRLWTYHGETPNATTDYSTT	···	PRSQES
	P6_ST3	DIW	···	MFRSNVSSEFQHKRT	···	FLKHYNSVWTFHGETPHATTDYSST	···	SRSQES
	P4_L26	DFW	···	MFKGSSQSNFSNRRT	···	MLKYGGRVWTFHGETPRATTDSSNT	···	PRSQES
	P8_Wa	DFW	···	MFRSSSQNEFYNRRT	···	ILKYGGRVWTFHGETPRATTDSSST	···	PRSQES
	P14_HAL1166	DRW	···	FIKLEKNGAYSQYST	···	WMKREGRVYWYAGTTPNASESYYLT	···	PRSQTE
P[III]	P9_K8	DRW	···	FIKLTPYGTYTQYST	···	WMKRDNRVYWYQGATPNASESYYLT	···	PQSQTA
	P25_Dhaka6	DRW	···	FIKLTKSGNYSQYSS	···	WIKRDGRVYWFDGTVPNSSDNYYLT	···	PRTQTD

C

Strain	100		150		170 190		215
MpR12	NKW	···	LIKTSADGKYTEVAP	···	IMKYSNAIYKFMGSTPNT--TLGFHSM	···	SRENQN
P16_EC	DRW	···	FLKTSVNGSYARYNI	···	VAKHTDYLYSYIGETPTAG-QAY-YAF	···	PWAQQS
P16_EL	NRW	···	FLKTSVNGSYARYNI	···	VAKHTDYLYSYIGETPTAG-QAY-YAF	···	PWAQQS
P16_EMcN	DRW	···	FLKTSTTGSYARYNI	···	VAKHTDYLYSYVGETPTAG-QAY-YAF	···	PWAQHS
P16_EW	NRW	···	FMKTPTTGSYVRYNI	···	VAKHTDNLYSYVGETPTAG-QAY-YSS	···	PWSQQS
P16_EDIM	NRW	···	FMKTPTTGSYVRYNI	···	VAKHTDNLYSYVGETPTAG-QAY-YSS	···	PWSQQS
P16_ETD_822	NRW	···	FMKTPTTGSYVRYNI	···	VAKHTDNLYSYVGETPTAG-QAY-YSS	···	PWSQQS
P16_EB-Po	DRW	···	FLKASTNGSYARYNI	···	VAKHTDRLYSYIRETPNAG-QAY-YAF	···	PWAQQS
P20_EHP	DRW	···	LAKTSLTGSYSQYGI	···	IMKRSGYLYTYSGETPDAI-TDY-YTT	···	PWAQEA
P3_LQ269	DRW	···	VVKTAQGGSYSQYGP	···	VMKHNGKIYTYNGETPNAT-TGY-YST	···	PRAEES
P3_LQ285	DRW	···	VVKTAQGGSYSQYGP	···	VMKHNGKIYTYNGETPNAT-TGY-YST	···	PRAEES
P3_LQ6	DRW	···	VVKTAQGGSYSQYGP	···	VMKHNGKIYTYNGETPNAT-TGY-YST	···	PRAEES
P3_KS-11-573	DRW	···	VVKTTQNGSYSQYGP	···	VMKYSNRIYTYGGETPNAT-TIY-YSA	···	PRAEES
P3_RA108	DRW	···	LVKNTQNGSYSQYGP	···	IMKYSGRMYTYNGETPNAT-VGY-YST	···	PRAEES
P3_LQ321	DRW	···	VVKTAQGGSYSQYGP	···	VMKHNGKIYTYNGETPNAT-TGY-YST	···	PRAEES
P45_WC179	NRW	···	IMKSSPTGTYSQHAT	···	VMKLDWQIWTYSGETPNAG-THG-YFT	···	SRSQED
P45_RA116	NRW	···	IMKSSPTGTYSQHAT	···	VMKLDWQLWTYSGETPNAG-THG-YFT	···	SRSQED
P13_SCLS-M	DIW	···	FRRRSQHDTYVLEGT	···	AMKYGAKLFTFIGDTPSAAPQEYGYET	···	PRLPRE
P46_WC272	DFW	···	LMKSTQGGNLQKTNE	···	VMKYNARLQLYTGRTPNAVVTS--QSI	···	PRSEES
P46_WC173	DFW	···	LMKSTQGGNLQKTNE	···	VMKYNARLQLYTGRTPNAVVTS--QSI	···	PRSEES
P46_RA61	DFW	···	LMKSTQGGNLQKTNE	···	VMKYNARLQLYTGRTPNAVVTS--QSI	···	PRSEES
P46_LW9	DFW	···	LMKSTQGGNLQKTNE	···	VMKYNARLQLYTGRTPNATITS--QSI	···	PRSEES

Fig. S2. Estimation of glycan-binding ability of the isolated RVAs. (A) Maximum-likelihood tree of VP8* genes of the isolated and representative RVA strains. using models of GTR+G+I with bootstrap values of 500 replicates. The isolated RVAs are indicated in red color. Strains consisting genogroups of P[I] to P[V] were tinted in gray respectively. Sialic acid-dependent strains were surrounded by a red dotted line. (B) Partial amino acid sequence alignment of VP8* of the isolated and representative RVA strains. Residues interacting with sialic acids are highlighted in blue. Residues interacting with mucin cores and LNFPI glycans are highlighted in green, and type A histo-blood group antigens are highlighted in pink. (C) Partial amino acid sequence alignment of VP8* of MpR12 and other rodent RVA strains. Residues interacting with sialic acids are highlighted in blue.

Fig. S3

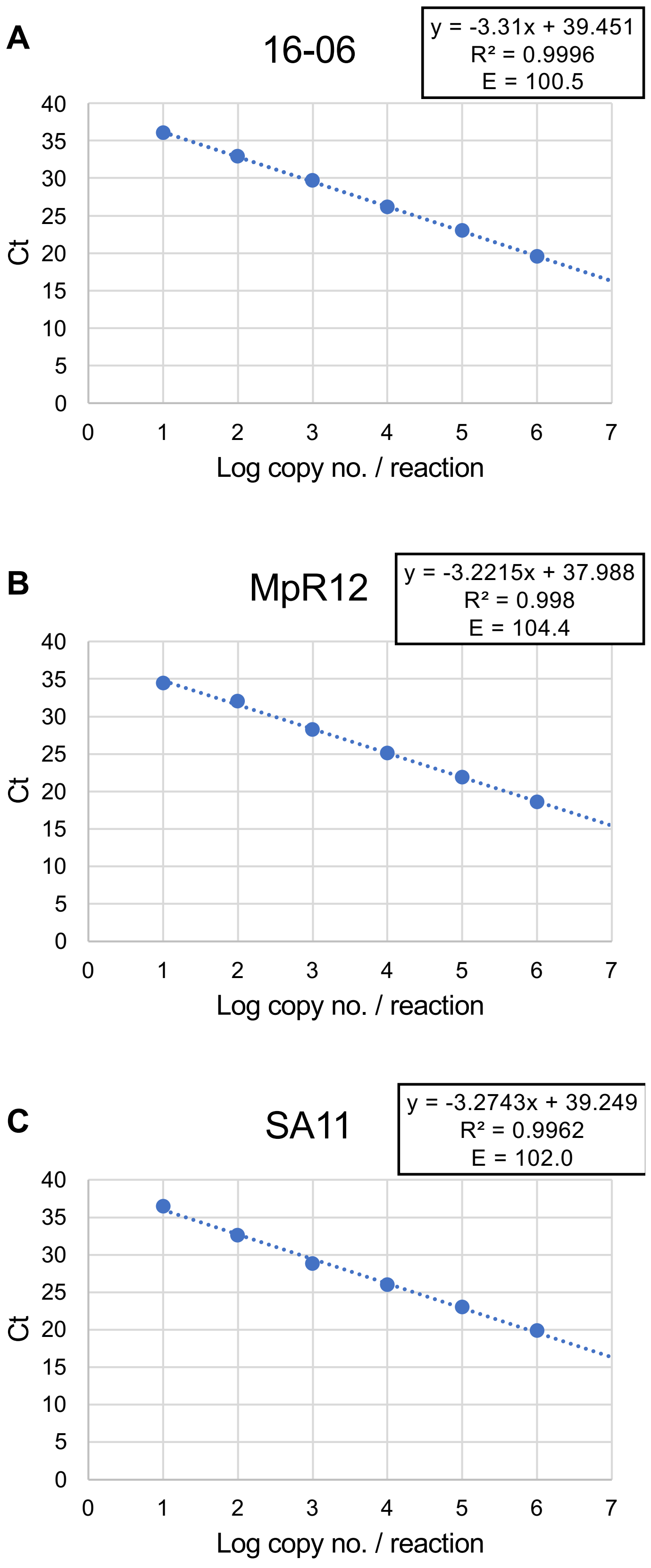

Fig. S3. Standard curves of qRT-PCR targeting 16-06 (A), MpR12 (B), and SA11 (C). Ct values were plotted against the log copy number of control plasmids. The regression curve (y), correlation coefficient (R^2), and PCR efficiency (E) were indicated, respectively.

Fig. S4

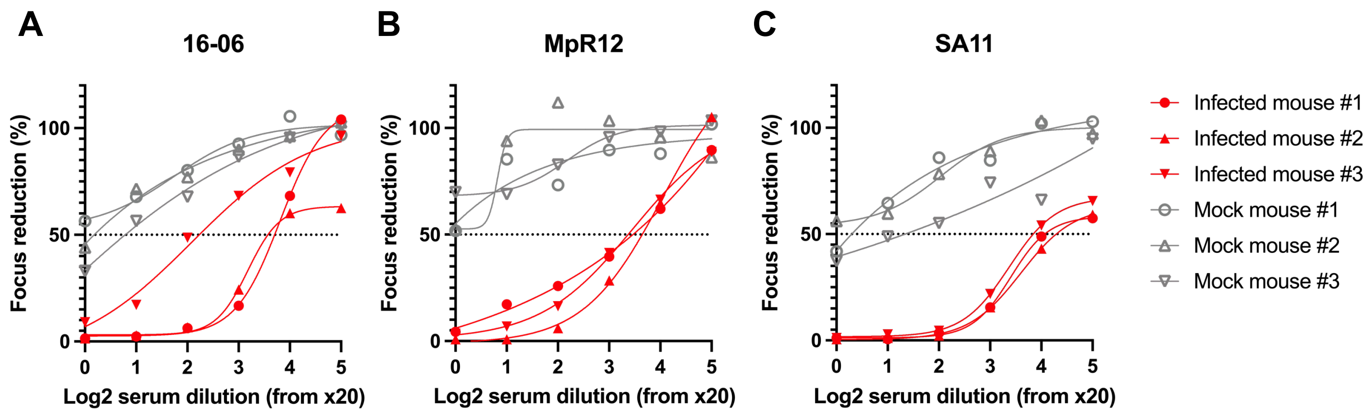

Fig. S4. Focus reduction neutralization test of RVA-infected suckling mice. Three-day-old BALB/c mice were orally inoculated with 1.0 × 10^5 FFU of RVA strains 16-06, MpR12, SA11 by gavage. Serum for FRNT were collected from suckling mice (*n*=3 in each group) at 15 days post infection (dpi) with RVAs. Percentages of focus reduction and interpolated sigmoidal fitting curves of 16-06 (A), MpR12 (B), and SA11 (C) were indicated.

Fig. S5

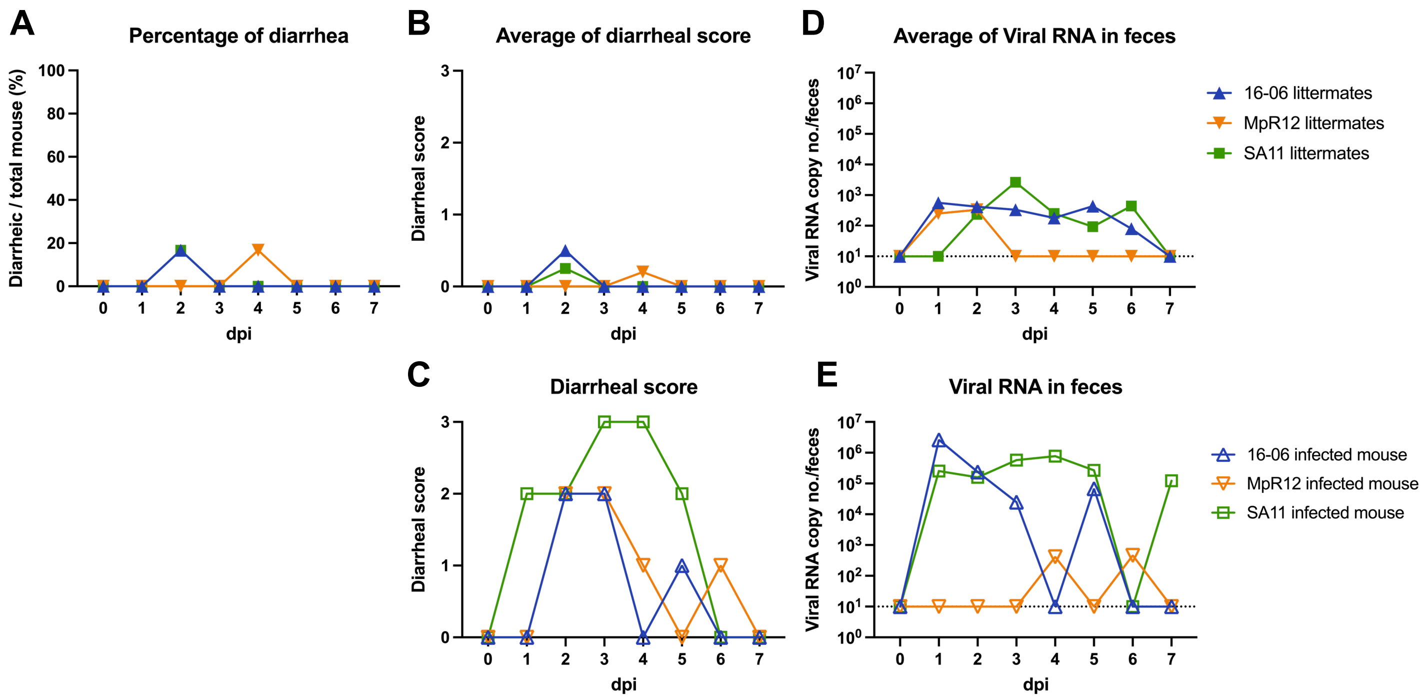

Fig. S5. Transmissibility of the isolated RVAs to uninfected litter suckling mice. One of littermates of three-day-old BALB/c mice were orally inoculated with 1.0×10^5 FFU of RVA strains 16-06, MpR12, SA11 by gavage (*n*=7 in each group). (A) Incidence rate of diarrhea of uninfected littermates in each group was monitored from 0 to 7 days post infection (dpi). (B and C) Fecal consistency of uninfected littermates (B) and infected mouse (C) in each group was scored according to the criteria described in the Methods. (D and E) Average of viral RNA copy numbers from the feces of uninfected littermates (D) and infected mouse (E) from 0 to 7 dpi were calculated based on the results of qRT-PCR. Dashed line indicates detection limit of qRT-PCR.
